# Response of Pseudomonas aeruginosa to the Innate Immune System-Derived Oxidants Hypochlorous Acid and Hypothiocyanous Acid

**DOI:** 10.1128/JB.00300-20

**Published:** 2020-12-18

**Authors:** Katie V. Farrant, Livia Spiga, Jane C. Davies, Huw D. Williams

**Affiliations:** a Department of Life Sciences, Imperial College London, London, United Kingdom; b National Heart and Lung Institute, Imperial College London, London, United Kingdom; c Department of Pediatric Respiratory Medicine, Royal Brompton Hospital, London, United Kingdom; Geisel School of Medicine at Dartmouth

**Keywords:** *Pseudomonas aeruginosa*, cystic fibrosis, efflux pumps, hypochlorous acid, hypothiocyanous acid, oxidative stress, peroxiredoxin

## Abstract

Pseudomonas aeruginosa is a significant nosocomial pathogen and is associated with lung infections in cystic fibrosis (CF). Once established, P. aeruginosa infections persist and are rarely eradicated despite host immune cells producing antimicrobial oxidants, including hypochlorous acid (HOCl) and hypothiocyanous acid (HOSCN). There is limited knowledge as to how P. aeruginosa senses, responds to, and protects itself against HOCl and HOSCN and the contribution of such responses to its success as a CF pathogen. To investigate the P. aeruginosa response to these oxidants, we screened 707 transposon mutants, with mutations in regulatory genes, for altered growth following HOCl exposure. We identified regulators of antibiotic resistance, methionine biosynthesis, catabolite repression, and PA14_07340, the homologue of the Escherichia coli HOCl-sensor RclR (30% identical), which are required for protection against HOCl. We have shown that RclR (PA14_07340) protects specifically against HOCl and HOSCN stress and responds to both oxidants by upregulating the expression of a putative peroxiredoxin, *rclX* (PA14_07355). Transcriptional analysis revealed that while there was specificity in the response to HOCl (231 genes upregulated) and HOSCN (105 genes upregulated), there was considerable overlap, with 74 genes upregulated by both oxidants. These included genes encoding the type 3 secretion system, sulfur and taurine transport, and the MexEF-OprN efflux pump. RclR coordinates part of the response to both oxidants, including upregulation of pyocyanin biosynthesis genes, and, in the presence of HOSCN, downregulation of chaperone genes. These data indicate that the P. aeruginosa response to HOCl and HOSCN is multifaceted, with RclR playing an essential role.

**IMPORTANCE** The bacterial pathogen Pseudomonas aeruginosa causes devastating infections in immunocompromised hosts, including chronic lung infections in cystic fibrosis patients. To combat infection, the host’s immune system produces the antimicrobial oxidants hypochlorous acid (HOCl) and hypothiocyanous acid (HOSCN). Little is known about how P. aeruginosa responds to and survives attack from these oxidants. To address this, we carried out two approaches: a mutant screen and transcriptional study. We identified the P. aeruginosa transcriptional regulator, RclR, which responds specifically to HOCl and HOSCN stress and is essential for protection against both oxidants. We uncovered a link between the P. aeruginosa transcriptional response to these oxidants and physiological processes associated with pathogenicity, including antibiotic resistance and the type 3 secretion system.

## Abstract (precis)

The bacterial pathogen Pseudomonas aeruginosa causes devastating infections in immunocompromised hosts, including chronic lung infections in cystic fibrosis patients. To combat infection, the host’s immune system produces the antimicrobial oxidants hypochlorous acid (HOCl) and hypothiocyanous acid (HOSCN). Little is known about how P. aeruginosa responds to and survives attack from these oxidants. To address this, we carried out two approaches: a mutant screen and transcriptional study. We identified the P. aeruginosa transcriptional regulator, RclR, which responds specifically to HOCl and HOSCN stress and is essential for protection against both oxidants. We uncovered a link between the P. aeruginosa transcriptional response to these oxidants and physiological processes associated with pathogenicity, including antibiotic resistance and the type 3 secretion system.

## INTRODUCTION

Pseudomonas aeruginosa is a leading cause of nosocomial infections that are life-threatening and difficult to treat (1). As an ESKAPE pathogen, it is one of the bacteria that poses the greatest public health threat (2). Furthermore, P. aeruginosa is the major pathogen associated with chronic lung infections in patients suffering from cystic fibrosis (CF) (3, 4). This genetic disease is caused by mutations in the cystic fibrosis transmembrane conductance regulator, which results in abnormal ion and water transport across epithelial membranes and leads to dehydrated airways and the establishment of respiratory infections (5). The immune response to infection is characterized by persistent neutrophil-dominated inflammation that is ineffective at clearing infection and results in progressive lung tissue damage (3, 4).

Host cells defend themselves against invading pathogens by the production of oxidants during the innate immune response, including the hypohalous acids: hypochlorous acid (HOCl), the active ingredient in bleach, and hypothiocyanous acid (HOSCN) (6). HOCl is a potent oxidant produced by neutrophils (7). The neutrophil oxidative burst that follows phagocytosis involves the reduction of O_2_ to superoxide (O_2_^−^) by the enzyme NADPH oxidase and the dismutation of O_2_^−^ to hydrogen peroxide (H_2_O_2_) (7). The heme enzyme myeloperoxidase (MPO) catalyzes the formation of HOCl from the reaction of H_2_O_2_ with chloride (Cl^−^) (7). MPO can also mediate H_2_O_2_ oxidation of other halides, including the pseudohalide thiocyanate (SCN^−^), to HOSCN. However, its primary physiological substrate in neutrophils is proposed to be HOCl, as the concentration of Cl^−^ in plasma (100 to 140 mM) is much greater than that of SCN^−^ (20 to 100 μM) (6, 8). The physiological concentration of HOCl is unknown due to the instability of this compound (6). At the epithelial cell surface of the lungs, HOSCN is produced through the concerted action of the dual oxidase (DUOX) and lactoperoxidase (LPO) (9). The epithelial cell DUOX releases H_2_O_2_ into the airway surface liquid, where LPO catalyzes the reaction of H_2_O_2_ with SCN^−^ to form HOSCN (9). HOCl can target proteins, lipids, DNA, and RNA; the most reactive targets of HOCl are thiol (S-H) groups of cysteine and methionine (7). Oxidation of cysteine by HOCl results in the formation of sulfenic acid, which can react with other cysteine thiols to form reversible disulfide bonds, and oxidation of methionine by HOCl results in the formation of methionine sulfoxide (6). Similar to HOCl, HOSCN reacts fastest with thiol groups of cysteine forming sulfenic acids or disulfide bonds (6). However, HOCl is a stronger oxidant than HOSCN, with rate constants for cysteine thiol groups of 3.2 × 10^7^ M^−1^ s^−1^ and 7.8 × 10^4^ M^−1^ s^−1^, respectively (6).

Despite HOCl and HOSCN having greater antibacterial activities than H_2_O_2_, studies of the bacterial mechanisms used to protect against oxidative stress have, until recent years, focused on the less potent oxidants, H_2_O_2_ and O_2_^−^ (6, 10). However, over the last 7 years, emerging studies into the bacterial response mechanisms to HOCl have identified thiol-based redox-sensing transcriptional regulators (11, 12). They include the Bacillus subtilis transcriptional factors OhrR, HypR, and PerR and the Escherichia coli transcriptional factors NemR, HypT, and RclR (13–17). HypT and RclR are the first described bacterial transcriptional regulators that are specific to sensing HOCl (16, 17); the others respond to a variety of stresses, including reactive electrophile species and organic hydroperoxides (11, 14, 15, 18–24). The identification of these HOCl-sensing regulators revealed that bacteria have specific mechanisms for responding to and protecting against HOCl stress and do not rely solely on mechanisms used against other oxidants. Virtually nothing is known about bacterial responses to HOSCN, apart from a study from Groitl et al. that investigated the transcriptional response of P. aeruginosa PA14 to HOSCN as well as to HOCl and another hypohalous acid, hypobromous acid (HOBr) (25). A key finding of this study was that all three oxidants caused protein aggregation and P. aeruginosa responded by increasing polyphosphate levels, which protected against protein aggregation and aided survival (25).

However, there is still very little known about how the clinically relevant pathogen P. aeruginosa senses, responds to, and protects against HOCl or HOSCN. Due to P. aeruginosa encountering these innate immune cell-derived oxidants in a variety of infection environments, including the neutrophilic environment of the CF lung, it is important to increase our knowledge of these specific oxidative stress responses.

Here, we have used a combination of mutant screening and transcriptomics to identify genes and systems used by P. aeruginosa to survive HOCl and HOSCN challenge. Our major finding is the identification of the P. aeruginosa RclR system that is specifically required for protection against both HOCl and HOSCN stress, in addition to discovering regulators with known roles in antibiotic resistance and methionine and carbon metabolism as having protective roles against HOCl.

## RESULTS

### Screening transposon mutants of P. aeruginosa regulatory genes for altered susceptibility to HOCl.

**FIG 1:**
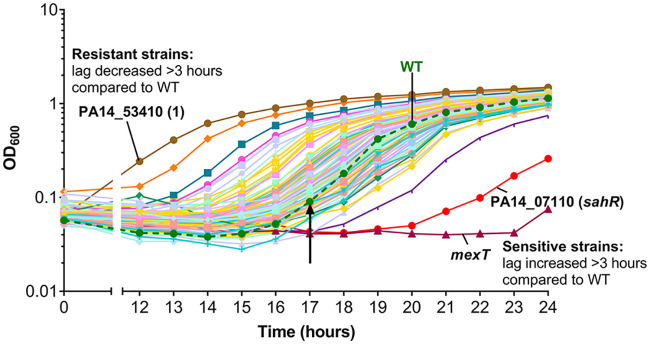
Identification of PA14 regulatory gene mutants with altered susceptibility to HOCl. Shown is an example screening plate with HOCl-sensitive (*mexT* and PA14_07110 [*sahR*]) and HOCl-resistant (PA14_53410 [1]) mutants labeled. Strains were grown in a 96-well format in LB medium with 4.4 mM HOCl, and optical density (OD_600_) was recorded as a measure of growth. Mutant strains that had an increased lag of >3 h compared to the WT were identified as HOCl sensitive, and strains with a decreased lag of >3 h compared to the WT were identified as HOCl resistant. The arrow represents the end of the WT lag phase. Strains grown in the absence of HOCl showed minor variations in growth, but none of the strains selected as sensitive or resistant (Table 1 and 2) had growth defects in LB only (data not shown).

We reasoned that targeting regulatory systems would be an effective way to uncover processes used by P. aeruginosa to protect against HOCl stress. To address this, we used mutants from the PA14 nonredundant transposon insertion mutant library (26), which contains 5,850 mutants from 4,596 genes (out of the 5,977 annotated genes) in the PA14 chromosome. To compile a subset of available mutants from this library, in genes classed as having confirmed or predicted regulatory roles, we used gene ontology information from the *Pseudomonas* genome database (27). A search for mutants in transcriptional regulator, two-component system, catalase, Ser/Thr kinase, sigma factor, and other regulatory genes resulted in a subset of 707 mutants (see Table S1 in the supplemental material). These 707 mutants were screened for altered susceptibility compared to the wild type (WT) when grown in the presence of 4.4 mM HOCl over 24 h. The most sensitive strains were identified as those that consistently failed to grow during the time of the assay or had an increased lag of >3 h compared to the WT. Resistant strains were identified as those that consistently had a decreased lag of >3 h compared to the WT. Figure 1 shows an example of a 96-well screening plate with HOCl-sensitive and HOCl-resistant mutants labeled. Identified HOCl-sensitive and HOCl-resistant mutants were rescreened, and those consistently displaying altered HOCl susceptibility are shown in Fig. S1. Statistical analysis of these data revealed 16 mutants that were significantly HOCl sensitive and 13 mutants that were significantly HOCl resistant compared to the WT at set time points (Tables 1 and 2).

**TABLE 1:** HOCl-sensitive regulatory gene mutants

PA14 locus tag and gene name^*a*^	PAO1 locus tag	Protein description	*P* value for mutant OD_600_ vs WT OD_600_ at set time points^*b*^		
			20 h	22 h	24 h
PA14_07110 *sahR*	PA0547	ArsR family transcriptional regulator	0.0420	0.0003	<0.0001
PA14_08780 *rpoC*	PA4269	DNA-directed RNA polymerase β chain	0.0280	0.0002	<0.0001
PA14_13660 *tpbA*	PA3885	Protein tyrosine phosphatase	0.0522	0.0006	0.0005
PA14_17900 *metR*	PA3587	LysR family transcriptional regulator	0.0342	0.0002	<0.0001
PA14_20730 *flgM* (1)	PA3351	Flagellar biosynthesis anti-sigma-28 factor	0.0288	0.0002	<0.0001
PA14_20730 *flgM* (2)	PA3351	Flagellar biosynthesis anti-sigma-28 factor	0.0299	0.0001	<0.0001
PA14_22370 (2)	PA3233	Hypothetical protein, cyclic nucleotide-binding domain	0.0274	0.0002	<0.0001
PA14_29740	PA2656	Two-component sensor	0.2349	0.0427	0.0214
PA14_32410 *mexT*	PA2492	LysR family transcriptional regulator of multidrug efflux	0.0370	0.0002	<0.0001
PA14_45950 *rsaL*	PA1431	Regulatory protein	0.1666	0.0367	0.0385
PA14_52570 *rsmA*	PA0905	Posttranscriptional global regulator	0.0340	0.0003	<0.0001
PA14_56620 *pyeR* (2)	PA4354	ArsR family transcriptional regulator	0.0396	0.0010	0.0068
PA14_60860 *nfxB*	PA4600	TetR family transcriptional regulator of multidrug efflux	0.0515	0.0049	0.0235
PA14_62490 *dksA*	PA4723	RNA polymerase-binding transcription factor	0.0088	0.0006	0.0018
PA14_68680 *envZ* (3)	PA5199	Two-component sensor	0.0494	0.0082	0.0714
PA14_70570 *recG*^*c*^	PA5345	ATP-dependent DNA helicase	0.0053	<0.0001	<0.0001

a Mutants were identified as HOCl sensitive by visual inspection (increased lag of >3 h compared to the WT) and statistical analysis of growth. The numbers in parentheses next to some of the gene mutants correspond to genes that have more than one mutant strain in the transposon library (Table S1).

b Statistical analysis performed on two biological replicates. *P* values were determined by one-way analysis of variance with Dunnett’s multiple-comparison test.

c *recG* is not a regulatory mutant *per se* but is on the same operon as *oxyR*, which encodes the H_2_O_2_-sensing transcriptional regulator; the transposon mutant in *oxyR* was not recoverable.

**TABLE 2:** HOCl-resistant regulatory gene mutants

PA14 locus tag and gene name^*a*^	PAO1 locus tag	Description	*P* value for mutant OD_600_ vs WT OD_600_ at set time points^*b*^			
			12 h	14 h	16 h	18 h
PA14_06950 (1)	PA0533	LuxR family transcriptional regulator	<0.0001	<0.0001	<0.0001	<0.0001
PA14_09760	PA4185	GntR family transcriptional regulator	0.9538	0.0098	0.0170	0.2154
PA14_10800 *ampR*	PA4109	LysR family transcriptional regulator of β-lactamase	<0.0001	<0.0001	<0.0001	0.0003
PA14_27900	PA2802	GntR family transcriptional regulator	0.1181	0.0040	0.0040	0.0442
PA14_38040 *cmrA*	PA2047	AraC family transcriptional regulator	<0.0001	<0.0001	<0.0001	<0.0001
PA14_44490 *anr*	PA1544	Transcriptional regulator of anaerobic metabolism	<0.0001	<0.0001	<0.0001	0.0053
PA14_50180 *fleR* (1)	PA1099	Two-component response regulator of flagellar biosynthesis	0.9990	0.2376	0.0471	0.3638
PA14_50200 *fleS* (2)	PA1098	Two-component sensor of flagellar biosynthesis	0.0610	<0.0001	0.0017	0.0481
PA14_56620 *pyeR* (1)	PA4354	ArsR family transcriptional regulator	<0.0001	<0.0001	<0.0001	0.0001
PA14_62530 *cbrA* (1)	PA4725	Two-component sensor of catabolic pathways	<0.0001	<0.0001	<0.0001	0.0398
PA14_62530 *cbrA* (2)	PA4725	Two-component sensor of catabolic pathways	0.8620	0.0482	0.0405	0.3687
PA14_62540 *cbrB*	PA4726	Two-component response regulator of catabolic pathways	0.0049	0.0003	0.0140	0.2098
PA14_68680 *envZ* (1)	PA5199	Two-component sensor	<0.0001	<0.0001	<0.0001	0.0015

a Mutants were identified as HOCl resistant by visual inspection (decreased lag of >3 h compared to the WT) and statistical analysis of growth. The numbers in parentheses next to some of the gene mutants correspond to genes that have more than one mutant strain in the transposon library (Table S1).

b Statistical analysis performed on two biological replicates. *P* values were determined by one-way analysis of variance with Dunnett’s multiple-comparison test.

### Links between antimicrobial resistance and HOCl susceptibility: the MexEF-OprN multidrug efflux pump protects P. aeruginosa from HOCl.

**FIG 2:**
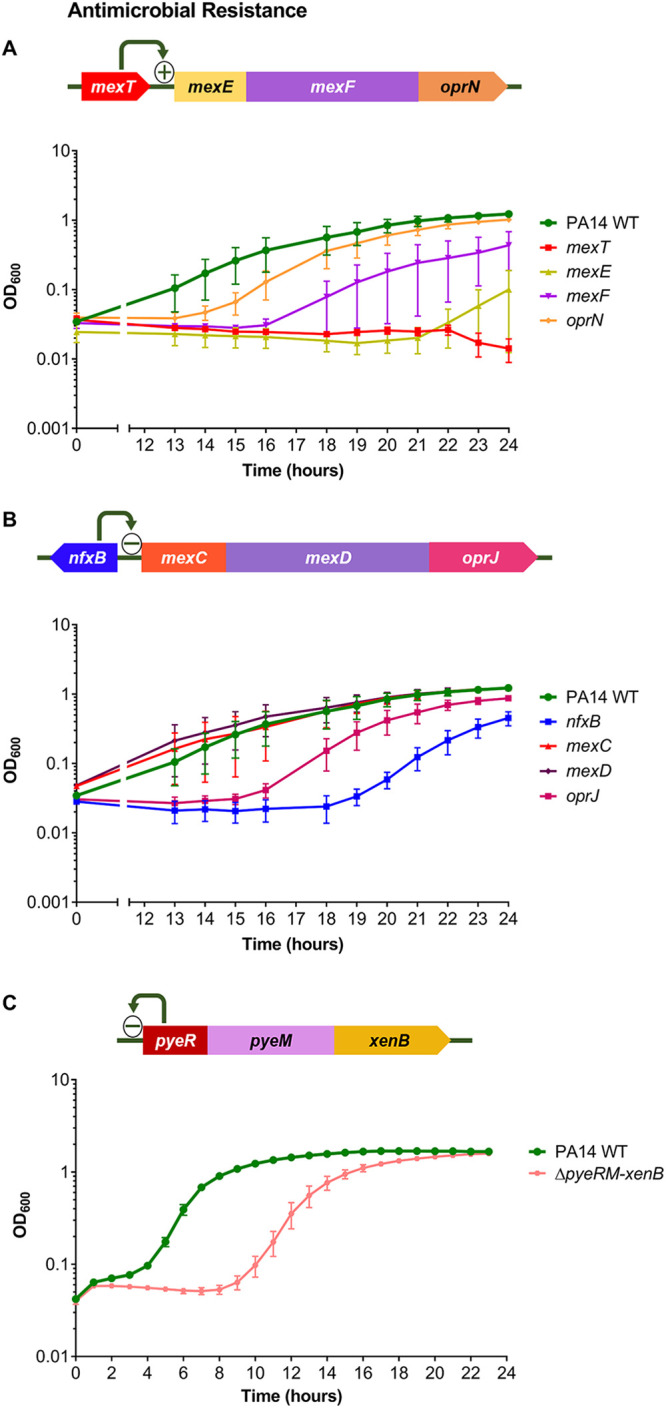
Mutants of genes involved in antimicrobial resistance displayed altered susceptibility to HOCl. (A) Gene arrangement of *mexT* and its adjacent *mexEF-oprN* operon. Growth of PA14 WT, *mexT*, *mexE*, *mexF*, and *oprN* strains in the presence of 4.4 mM HOCl. (B) Gene arrangement of *nfxB* and its adjacent *mexCD-oprJ* operon. Growth of PA14 WT, *nfxB*, *mexC*, *mexD*, and *oprJ* strains in the presence of 4.4 mM HOCl. For *mexC* and *mexD*, 2 transposon mutants of each gene were available and showed similar phenotypes; therefore, only 1 mutant of each is displayed. (C) Gene arrangement of the *pyeRM-xenB* operon; PyeR autorepresses the *pyeRM-xenB* operon (33). Growth of PA14 WT and Δ*pyeRM-xenB* strains in the presence of 5.1 mM HOCl. Graphs display the means and standard errors of the means for three independent experiments (A and B) and three biological replicates (C), representative of three independent experiments. The plus and minus signs in the gene arrangement diagrams represent positive and negative regulators, respectively. All mutant strains displayed statistically significant altered susceptibility to HOCl compared with the WT at different time points, apart from *oprN*, *mexC*, *mexD*, and *oprJ* (*P < *0.05; one-way analysis of variance with Dunnett’s multiple-comparison test). Strains grown in the absence of HOCl showed no growth defects (data not shown). All transposon mutants and the in-frame deletion were confirmed by PCR.

A mutant in *mexT* was unable to grow in the presence of HOCl (Fig. 2A). MexT is a LysR-type regulator that positively regulates expression of its adjacent operon *mexEF-oprN*, which encodes a multidrug efflux pump (28, 29). MexT regulates a number of genes (30, 31), in addition to the *mexEF-oprN* operon; thus, to determine whether loss of the efflux pump is associated with HOCl sensitivity, transposon mutants of all three efflux pump genes were tested for altered HOCl susceptibility. They displayed increased sensitivity in the order of *mexE* > *mexF* > *oprN* (Fig. 2A). For all three mutant strains, the transposon is inserted in the middle of the gene. This suggests that the MexEF-OprN efflux pump provides P. aeruginosa with protection against HOCl. MexT positively regulates 12 other genes (30) in addition to *mexEF-oprN* and *pyeR* (mentioned below); mutants in 8 of these were available for screening, and 4 displayed HOCl sensitivity (PA14_41990, PA14_39060, PA14_27755, and PA14_27770), although none were as sensitive as *mexE* (Table S2).

Another multidrug efflux pump regulator, NfxB, was required for protection against HOCl, as an *nfxB* mutant had increased HOCl sensitivity (Fig. 2B). NfxB is a negative regulator of the *mexCD-oprJ* multidrug efflux operon (32), and transposon mutants in these genes were tested; only *oprJ* had altered susceptibility, displaying HOCl sensitivity (Fig. 2B).

MexT regulates the *pyeRM-xenB* operon, in which *pyeR* encodes an ArsR family repressor, *pyeM* encodes a major facilitator superfamily (MFS) transporter, and *xenB* encodes an uncharacterized xenobiotic reductase (33). Transposon mutants in *pyeR* showed variable susceptibility to HOCl (Fig. S1); therefore, we constructed a whole-operon in-frame Δ*pyeRM-xenB* deletion mutant, which displayed HOCl sensitivity (Fig. 2C). This suggests a role for this MexT-regulated operon in protecting P. aeruginosa against HOCl.

### Metabolic regulators are involved in protection against HOCl.

**FIG 3:**
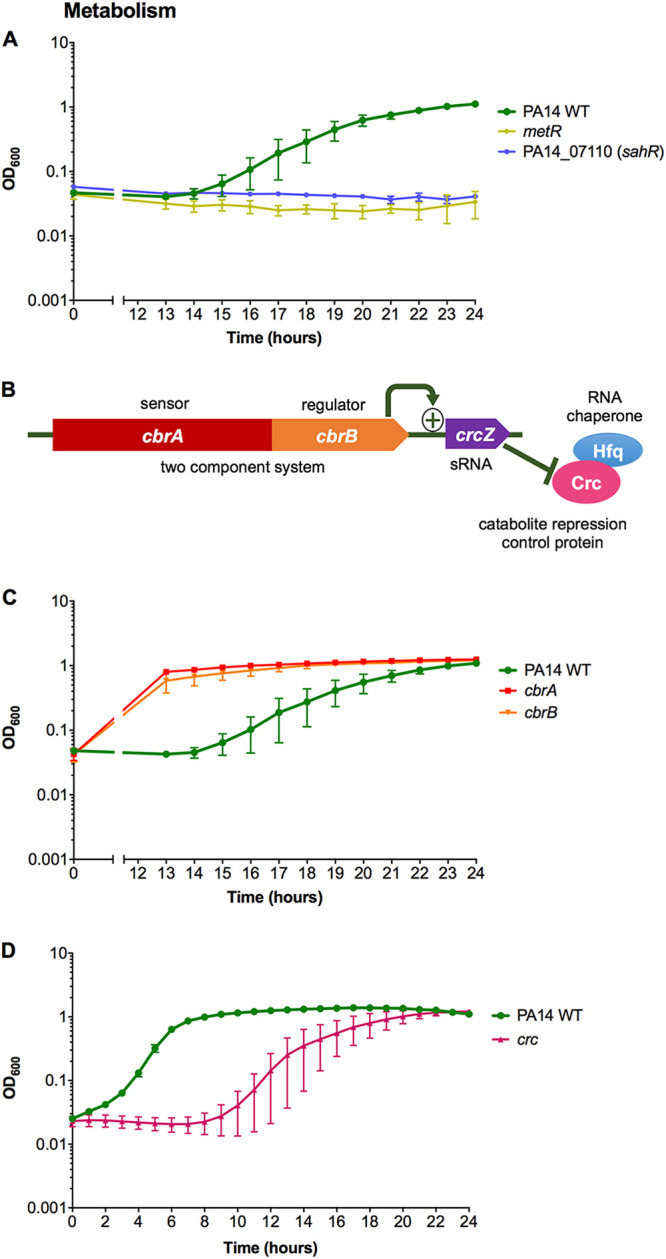
Mutants of genes involved in metabolism displayed altered susceptibility to HOCl. (A) Growth of PA14 WT, *metR*, and PA14_07110 (*sahR*) strains in the presence of 4.4 mM HOCl. (B) Gene arrangement of the two-component system *cbrAB* that regulates the expression of the sRNA CrcZ, which relieves Crc/Hfq-mediated repression of target mRNAs. (C and D) Growth of PA14 WT, *cbrA*, and *cbrB* strains (C) and PA14 WT and *crc* strains (D) in the presence of 4.4 mM HOCl. For *cbrA*, 2 transposon mutants were available and showed similar phenotypes; therefore, only 1 mutant is displayed. Graphs display the means and standard errors of the means for three independent experiments (A and C) and three biological replicates (D), representative of three independent experiments. The plus sign in the gene arrangement diagram represents a positive regulator. All mutant strains displayed statistically significant altered susceptibility to HOCl compared with the WT at different time points (*P < *0.05; one-way analysis of variance with Dunnett’s multiple-comparison test). Strains grown in the absence of HOCl showed no growth defects (data not shown). All transposon mutants were confirmed by PCR.

A mutant in *metR* that encodes a regulator homologous (40% identity) to E. coli MetR, a regulator of methionine biosynthesis genes (34), was HOCl sensitive (Fig. 3A). A mutant in PA14_07110 (*sahR*) was also HOCl sensitive (Fig. 3A). This gene encodes an ortholog of SahR from *Desulfovibrio* spp., which is a transcriptional regulator of the *S*-adenosylmethionine cycle genes that are involved in methionine recycling (35).

The CbrA/B two-component system is involved in controlling carbon and nitrogen metabolism and carbon catabolite repression (36–38). CbrA/B acts through activation of the small RNA (sRNA) CrcZ, which sequesters the RNA chaperone Hfq and the catabolite repression control protein Crc from mRNA target genes, relieving catabolite repression (38–40) (Fig. 3B). Mutants of *cbrAB* were HOCl resistant (Fig. 3C), and subsequently the *crc* transposon mutant was tested and found to be HOCl sensitive (Fig. 3D). These phenotypes correlate with the opposing regulatory roles of CbrA/B and Crc.

### Regulators with putative roles in the oxidative stress response are required for protection against HOCl.

**FIG 4:**
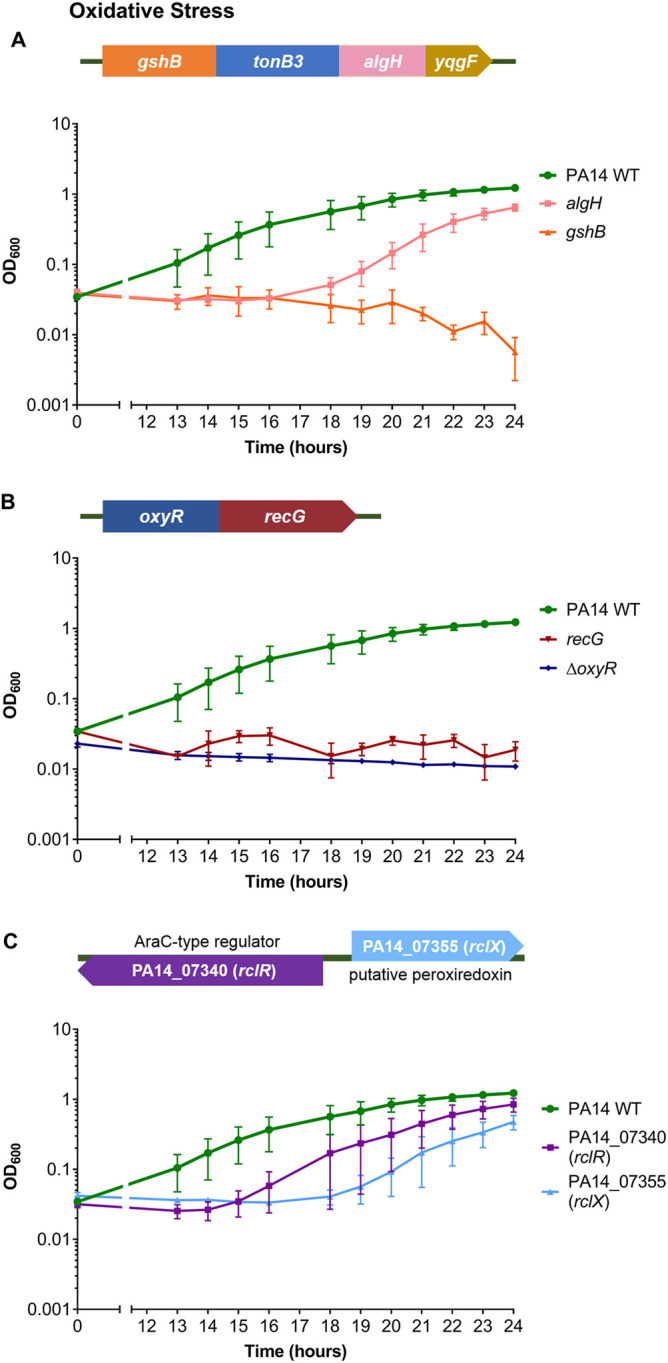
Mutants of genes involved in oxidative stress defense displayed altered susceptibility to HOCl. (A) Gene arrangement of the predicted 4-gene operon, *gshB-tonB3-algH-yqgF*. Growth of PA14 WT, *algH*, and *gshB* strains in the presence of 4.4 mM HOCl. PA14 transposon mutants were not available for *yqgF* but were for *tonB3*; however, they did not display altered HOCl susceptibility (data not shown). (B) Gene arrangement of the *oxyR-recG* operon. Growth of PA14 WT, *recG*, and Δ*oxyR* strains in the presence of 4.4 mM HOCl. (C) Gene arrangement of PA14_07340 (*rclR*) and PA14_07355 (*rclX*). Growth of PA14 WT, PA14_07340 (*rclR*), and PA14_07355 (*rclX*) strains in the presence of 4.4 mM HOCl. Graphs display the means and standard errors of the means for three independent experiments (two independent experiments only for *recG*). All mutant strains displayed statistically significant altered susceptibility to HOCl compared with the WT at different time points (*P < *0.05; one-way analysis of variance with Dunnett’s multiple-comparison test). Strains grown in the absence of HOCl showed no growth defects (data not shown). All transposon mutants and the in-frame deletion were confirmed by PCR.

A mutant in the *algH* gene, which encodes a hypothetical protein that has been crystallized and predicted to be a potential redox sensor, was HOCl sensitive (Fig. 4A) (41). *algH* is part of a 4-gene operon that includes *gshB*, which encodes a glutathione synthetase; a *gshB* mutant was tested, and it displayed HOCl sensitivity (Fig. 4A).

A mutant in *oxyR* encoding an H_2_O_2_-sensing transcriptional regulator (42) was nonviable; however, a mutant was available for its adjacent gene, a putative DNA repair enzyme, *recG* (42), and was HOCl sensitive (Fig. 4B). The sensitivity of the *recG* mutant to HOCl was not due to disruption of *oxyR*, as reverse transcription-PCR (RT-PCR) confirmed the *oxyR* transcript was present in the *recG* mutant (data not shown). We constructed an in-frame deletion of *oxyR*, which displayed HOCl sensitivity (Fig. 4B). This suggests that the *oxyR-recG* operon is involved in protection against HOCl as well as H_2_O_2_ (42).

P. aeruginosa mutants of homologues of other transcriptional regulators that respond to HOCl in bacteria, NemR (PA14_36300), HypT (PA14_71640), and OhrR (PA14_27230), did not display consistent HOCl-sensitive or -resistant phenotypes in the screen (Fig. S2). However, a mutant in PA14_07340, the P. aeruginosa homologue of the E. coli reactive chlorine-specific transcriptional regulator, RclR (17), was identified as HOCl sensitive (Fig. 4C). The *rclR* mutant was one of three strains from the screen that showed consistent, reproducible sensitivity to HOCl and displayed significant altered growth in the presence of the oxidant when rescreened (Fig. 4C) but did not show significant altered growth at the time points analyzed in the initial screen (Table 1). P. aeruginosa *rclR* (PA14_07340) is adjacent to a single-gene operon, PA14_07355, that encodes a putative peroxiredoxin enzyme of the alkylhydroperoxidase (AhpD) family (Fig. 4C). A mutant in PA14_07355 was tested and displayed HOCl sensitivity (Fig. 4C).

### P. aeruginosa RclR is specifically required for protection against HOCl and HOSCN.

**FIG 5:**
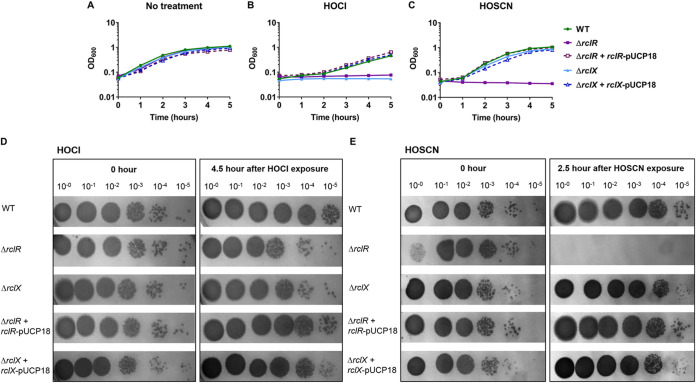
Mutants of *rclR* and its adjacent gene *rclX* are sensitive to HOCl, and *rclR* is sensitive to the epithelium-derived oxidant HOSCN. (A to C) Growth of PA14 WT, Δ*rclR*, Δ*rclR* + *rclR*-pUCP18, Δ*rclX*, and Δ*rclX* + *rclX*-pUCP18 strains in LB medium (A), LB medium with 4.4 mM HOCl (B), or LB medium with 0.8 mM HOSCN (C). Graphs display the means and standard errors of the means for three biological replicates, representative of three independent experiments. For HOSCN sensitivity assays, a control reaction of glucose oxidase, glucose, and potassium thiocyanate was carried out, which resulted in the production of H_2_O_2_ only; the Δ*rclR* strain was not sensitive under these conditions, confirming sensitivity is due to HOSCN production (data not shown). The addition of the empty pUCP18 vector to Δ*rclR* and Δ*rclX* strains had no effect on the susceptibility of these mutants to HOCl or HOSCN (data not shown). (D and E) Viability CFU assays. Mid-exponential-phase cultures of PA14 WT, Δ*rclR*, Δ*rclX*, Δ*rclR* + *rclR*-pUCP18, and Δ*rclX* + *rclX*-pUCP18 strains were incubated in LB medium containing 4.4 mM HOCl (D) or 0.8 mM HOSCN (E). Samples were taken immediately after oxidant exposure and after 4.5 h (HOCl) or 2.5 h (HOSCN), diluted in PBS, spot titered onto LBA, and incubated at 37°C overnight or until CFU could be visualized. (E) For undiluted (10^−0^) Δ*rclR* samples taken immediately after HOSCN exposure (0 h), fewer viable cells are visualized because HOSCN acted immediately, whereas in the other dilution factors HOSCN was diluted down and, therefore, did not affect growth. Images are representative of three independent experiments.

To confirm the role of P. aeruginosa RclR in protection against HOCl, in-frame deletion mutants of *rclR* and the putative peroxiredoxin gene, which we named *rclX*, were constructed. The susceptibility of the mutants to HOCl was retested; Δ*rclR* and Δ*rclX* strains grew similarly to the WT in the absence of oxidant (Fig. 5A), but neither mutant grew in the presence of HOCl over the time course of the experiment (Fig. 5B). Complementation of Δ*rclR* and Δ*rclX* strains with *rclR*-pUCP18 and *rclX*-pUCP18, respectively, restored growth in the presence of HOCl to that of the WT (Fig. 5B). To test the specificity of RclR, mutants were exposed to other reactive oxygen species (H_2_O_2_, the O_2_^−^ generator methyl viologen, and *tert*-butyl hydroperoxide [TBH]), reactive electrophilic species (*N*-ethylmaleimide [NEM], methylglyoxal, and diamide), and the nitric oxide generator diethylamine NONOate (DEANO). These reactive species did not alter the growth of the Δ*rclR* strain compared to the WT, indicating that P. aeruginosa RclR is not required for protection against these compounds (Fig. S3). Due to HOSCN being another physiologically relevant, thiol-reactive oxidant produced by the immune system (9), we were interested in determining whether Δ*rclR* and Δ*rclX* strains had altered sensitivity to this oxidant. Growth of Δ*rclR* and Δ*rclX* strains in the presence of 0.8 mM HOSCN revealed that the Δ*rclR* strain was sensitive to HOSCN, whereas the Δ*rclX* strain was not at this concentration (Fig. 5C). Complementation of the Δ*rclR* strain with *rclR*-pUCP18 restored growth in the presence of HOSCN (Fig. 5C). These data were supported by viability assays, which showed HOCl inhibited growth of Δ*rclR* and Δ*rclX* strains (Fig. 5D), and HOSCN was bactericidal toward the Δ*rclR* strain (Fig. 5E). When the HOSCN concentration was increased to 1 mM, the Δ*rclX* as well as the Δ*rclR* strain displayed sensitivity to this oxidant (Fig. S4). Therefore, both RclR and RclX are required for protection against HOCl and HOSCN.

The sensitivity assays thus far were performed on planktonic cells in Luria-Bertani (LB) medium; to determine whether the findings were consistent when cells were grown under conditions relevant to the CF lung environment, sensitivity assays for Δ*rclR* and Δ*rclX* mutants were repeated in ASM (artificial sputum medium), which mimics the CF sputum (43, 44). Both Δ*rclR* and Δ*rclX* strains displayed HOCl and HOSCN sensitivity when grown in ASM (Fig. S5), confirming the findings of the LB assays. Additionally, during chronic CF lung infections, P. aeruginosa persists in biofilms. To determine whether P. aeruginosa mutants display similar HOCl and HOSCN susceptibility when forming biofilms, Δ*rclR* and Δ*rclX* mutants were grown in a suspended ASM biofilm assay in the absence or presence of HOCl and HOSCN (adapted from reference 44). The WT, Δ*rclR*, and Δ*rclX* strains formed biofilms in ASM in the absence of the oxidants (Fig. S6). However, in the presence of 3.5 mM HOCl and 0.53 mM HOSCN, both Δ*rclR* and Δ*rclX* strains were unable to grow and appeared sensitive to these oxidants compared to the WT, which was able to form biofilms at these concentrations (Fig. S6). These data confirm the relevance of the impact of HOCl and HOSCN on P. aeruginosa in a more realistic CF environment.

### RclR regulates induction of *rclX* expression in response to HOCl and HOSCN stress.

**FIG 6:**
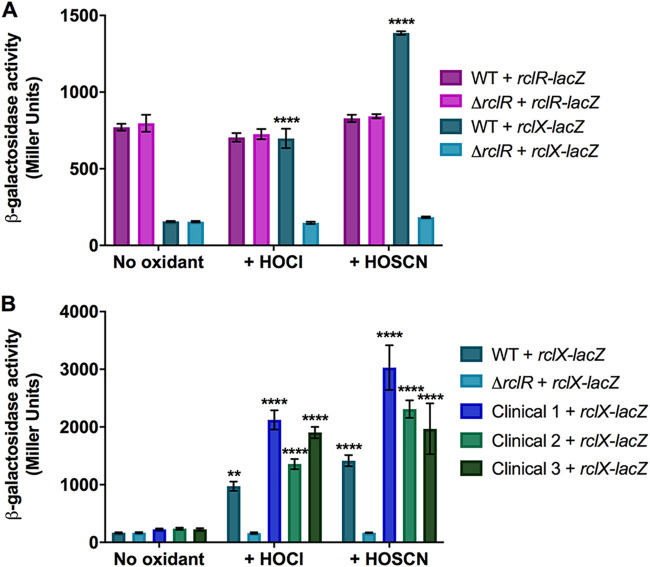
HOCl and HOSCN stress induces RclR-dependent regulation of *rclX* expression in PA14 P. aeruginosa and clinical CF isolates. (A) Activity of *rclR-lacZ* and *rclX-lacZ* transcriptional fusions in the PA14 WT and Δ*rclR* strains in the absence or presence of 2.2 mM HOCl or 0.8 mM HOSCN. (B) Activity of the *rclX-lacZ* transcriptional fusions in WT, Δ*rclR*, and clinical CF isolates 1, 2, and 3 in the absence or presence of 2.2 mM HOCl or 0.8 mM HOSCN. Strains were grown aerobically in LB medium until mid-exponential phase, when HOCl, HOSCN, or LB only was added, and samples were taken for β-galactosidase activity measurements at 30 min. The concentrations of oxidants used caused a 30- to 120-min lag in growth of exponential-phase cultures. PA14 WT and Δ*rclR* strains carrying the empty pMP220 vector expressed low β-galactosidase activity (∼100 Miller units) under all of the treatment conditions (data not shown). Data show the means and standard errors of the means for three biological replicates, representative of three independent experiments (six biological replicates for untreated strains [B]). Statistical analysis was performed using one-way analysis of variance with Tukey’s multiple-comparison *post hoc* test. **, *P < *0.01; ****, *P < *0.0001; compared to the same strain untreated (no oxidant).

To determine whether RclR regulates expression of itself and/or *rclX* under HOCl and HOSCN stress, transcriptional *rclR-lacZ* and *rclX-lacZ* fusions were constructed and introduced into the WT and Δ*rclR* strains for *in vivo* β-galactosidase assays. The activity of *rclX-lacZ* increased following HOCl and HOSCN exposure in the WT strain but not in the Δ*rclR* strain, suggesting that expression of *rclX* increases following HOCl and HOSCN stress dependent on RclR regulation (Fig. 6A). No difference in the activity of *rclR-lacZ* was observed between the WT and Δ*rclR* strains in the absence or presence of HOCl and HOSCN, suggesting RclR does not autoregulate its own expression and is constitutively expressed (Fig. 6A). We next examined if the activation of *rclX* in response to these oxidants occurred in clinical CF isolates of P. aeruginosa. The activity of *rclX-lacZ* in three CF isolates was measured in the absence or presence of HOCl and HOSCN. The *rclX* gene was significantly induced in response to both oxidants in all three clinical isolates compared to untreated controls (Fig. 6B). This confirmed that *rclX* induction appears to be an important adaptive response to these oxidants across P. aeruginosa laboratory and clinical strains.

To investigate the possible function of RclX, we performed homology modeling that fitted the RclX sequence with 32.1% sequence identity to the AhpD-like protein, Lpg0406, from Legionella pneumophila, which belongs to the carboxymuconolactone decarboxylase family (45) (Fig. S7). The predicted structure was a homohexamer (Fig. S7A), formed from monomers with an AhpD-like fold containing six α-helices and a conserved catalytic CXXC motif (Fig. S7B). RclX was sequence aligned with Lpg0406, PA0269, another AhpD-like protein from P. aeruginosa (46), and MtAhpD, from Mycobacterium tuberculosis (47–49) (Fig. S7C). Four out of the five catalytic residues of MtAhpD responsible for peroxidase activity (47, 48) were conserved in RclX (Fig. S7C), suggesting a role for RclX in oxidant detoxification.

### Transcriptional changes in PA14 WT and Δ*rclR* strains in response to HOCl and HOSCN exposure.

**FIG 7:**
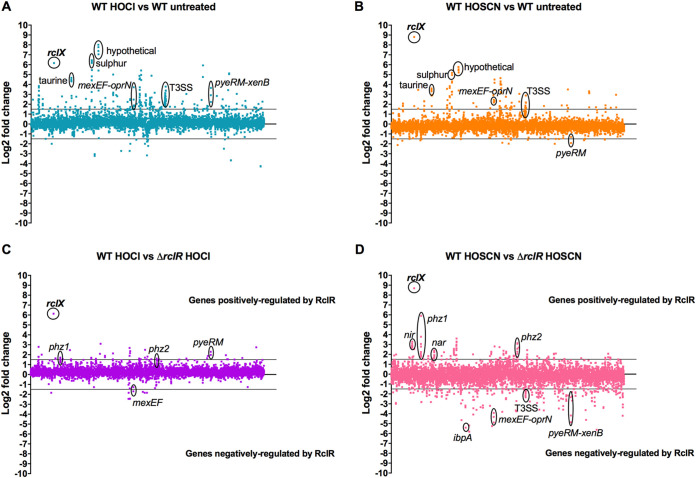
Relative changes in gene expression in PA14 WT and Δ*rclR* strains in the presence of HOCl or HOSCN. Log_2_ fold change of gene expression was plotted against the gene locus tag in order of genomic location. (A) Effect of HOCl on P. aeruginosa gene expression, WT plus HOCl versus WT untreated. (B) Effect of HOSCN on P. aeruginosa gene expression, WT plus HOSCN versus WT untreated. (C) Identification of genes regulated by RclR in the presence of HOCl, WT plus HOCl versus Δ*rclR* strain plus HOCl. (D) Identification of genes regulated by RclR in the presence of HOSCN, WT plus HOSCN versus Δ*rclR* strain plus HOSCN. Lines indicate cutoff values at 1.5 or −1.5 log_2_ fold change. (A and B) Highlighted upregulated genes include *rclX*, hypothetical operon (PA14_21570-PA14_21580-PA14_21590-PA14_21600), sulfur transport genes (*ssuD*-PA14_19570-*ssuB*), taurine transport genes (PA14_12920-PA14_12940-PA14_12960), drug efflux pump operon *mexEF-oprN*, *pyeRM-xenB* operon (HOCl), and T3SS genes. Downregulated genes include *pyeRM* (HOSCN). (C and D) In these plots, positive log_2_ fold change values indicate genes positively regulated by RclR, and negative log_2_ fold change values indicate genes negatively regulated by RclR. Highlighted genes positively regulated include *rclX*, pyocyanin biosynthesis operons *phz1* and *phz2*, denitrification *nir* and *nar* operons (HOSCN), and *pyeRM* (HOCl). Negatively regulated genes include *pyeRM-xenB* (HOSCN), *mexEF-oprN*, T3SS genes (HOSCN), and heat shock protein *ibpA* (HOSCN).

The response of P. aeruginosa to HOCl and HOSCN was further characterized by measuring changes in gene expression in the WT and Δ*rclR* strains. PA14 WT and Δ*rclR* exponential-phase cultures were exposed to 2.2 mM HOCl or 0.8 mM HOSCN, and gene expression was analyzed by transcriptome sequencing (RNA-seq). Genes with a log_2_ fold change of >1.5 were considered upregulated, and genes with a log_2_ fold change of <−1.5 were considered downregulated (Fig. 7). All transcriptomic data are presented in Data Set S1 and are accessible through the Gene Expression Omnibus (GEO) database under accession number GSE124385. For identification of genes whose expression is altered in the presence of HOCl or HOSCN, expression was compared in treated WT strains versus untreated WT strains. HOCl upregulated 231 genes and downregulated 20 genes in the WT strain (Fig. 7A). HOSCN upregulated 105 genes and downregulated 16 genes in the WT strain (Fig. 7B). The importance of *rclX* in the P. aeruginosa response to HOCl and HOSCN was confirmed, as it was strongly upregulated with a >6 and >8 log_2_ fold change, respectively, and this was consistent with the gene fusion expression data (Fig. 6 and 7A and B). These data have been validated by quantitative RT-PCR (qRT-PCR) for a subset of the most highly upregulated and downregulated genes (including *rclX*) whose patterns of expression were confirmed (5 upregulated and 1 downregulated) (Fig. S8). For the identification of genes whose expression is dependent on RclR, expression was compared in treated WT strains versus treated Δ*rclR* strains. Therefore, in Fig. 7C and D, genes displaying increased expression indicate genes positively regulated by RclR, and genes displaying decreased expression indicate genes negatively regulated by RclR. In the presence of HOCl, RclR upregulated 42 genes and downregulated 7 genes (Fig. 7C), and in the presence of HOSCN, RclR upregulated 132 genes and downregulated 213 genes (Fig. 7D). Regulation of *rclX* by RclR was confirmed, as it was the gene most strongly upregulated by RclR in the presence of HOCl and HOSCN (Fig. 7C and D). Expression of *rclR* was not altered following exposure to HOCl or HOSCN, agreeing with our previous gene fusion data (Fig. 6A and Data Set S1). Furthermore, the genes of homologues of other HOCl-responsive transcriptional regulators from E. coli and B. subtilis, NemR (PA14_36300), HypT (PA14_71640), and OhrR (PA14_27230), did not have altered expression in response to HOCl or HOSCN (Data Set S1).

The upregulated and downregulated genes were examined for overlap between the response to both oxidants and were categorized into functional groups based on assigned gene ontologies in the *Pseudomonas* genome database (27) (Fig. S9). Of the 105 genes upregulated in response to HOSCN, 74 (70%) were also upregulated in response to HOCl, indicating similarities between the bacterial responses to these two oxidants (Fig. S9A). The functional groups that displayed the largest percentage of genes upregulated in response to HOCl and HOSCN were noncoding RNA, protein secretion/export apparatus, and antibiotic resistance and susceptibility (Fig. S9B). Of the 132 RclR-regulated genes whose expression was upregulated in the presence of HOSCN, only 12 (9%) of those were also upregulated in the presence of HOCl (Fig. S9C), and of the 213 RclR-regulated genes downregulated following HOSCN exposure, only 2 (1%) were downregulated after HOCl exposure (Fig. S9D). For RclR-regulated genes, the functional group with the highest percentage of genes upregulated following HOCl and HOSCN exposure was secreted factors (Fig. S9E). RclR downregulated a large number of genes in the presence of HOSCN, particularly those in the chaperone and heat shock protein functional groups (Fig. S9F). The observed regulatory effects might be a direct consequence of loss of RclR-mediated regulation or a result of secondary pathways impacted by *rclR* deletion.

### tRNA, type 3 secretion system (T3SS), sulfur and taurine metabolism, and MexT-regulated efflux pump genes show increased expression following exposure to HOCl and HOSCN.

**FIG 8:**
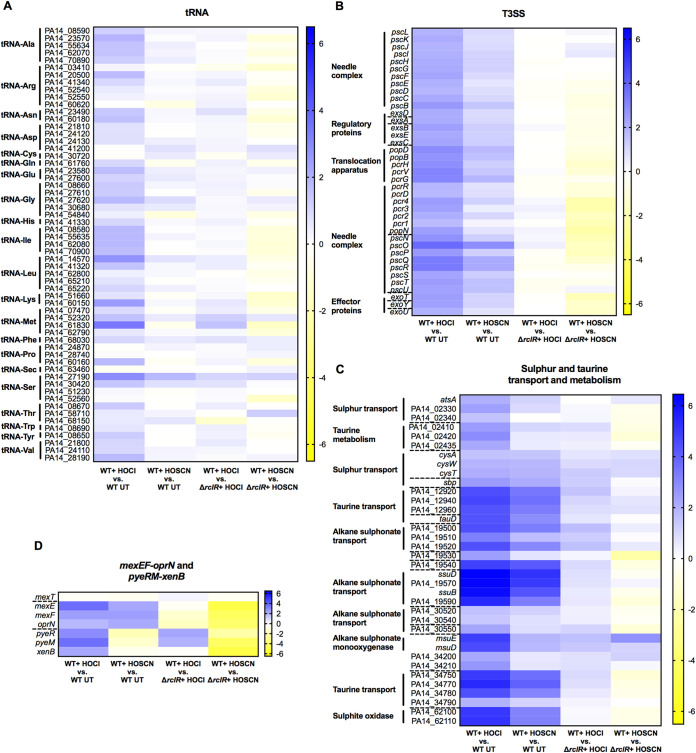
Heat maps displaying expression of tRNA, T3SS, sulfur and taurine transport and metabolism, and *mexEF-oprN* efflux pump and *pyeRM-xenB* genes in PA14 WT and Δ*rclR* strains after HOCl or HOSCN exposure. (A) tRNAs. (B) T3SS. (C) Sulfur and taurine transport and metabolism. (D) *mexEF-oprN* and *pyeRM*-xenB. Expression of genes is color coordinated from 6.5 log_2_ fold change (blue) to −6.5 log_2_ fold change (yellow). The first column indicates HOCl-responsive genes (log_2_ fold change of WT plus HOCl versus WT untreated [UT]), and the second column indicates HOSCN-responsive genes (log_2_ fold change of WT plus HOSCN versus WT UT). The third column indicates RclR-regulated genes under HOCl stress (log_2_ fold change of WT plus HOCl versus Δ*rclR* strain plus HOCl), and the fourth column indicates RclR-regulated genes under HOSCN stress (log_2_ fold change of WT plus HOSCN versus Δ*rclR* strain plus HOSCN). In these columns, genes that are positively or negatively regulated are indicated by blue or yellow, respectively. Broken lines indicate the start and end of operons.

**FIG 9:**
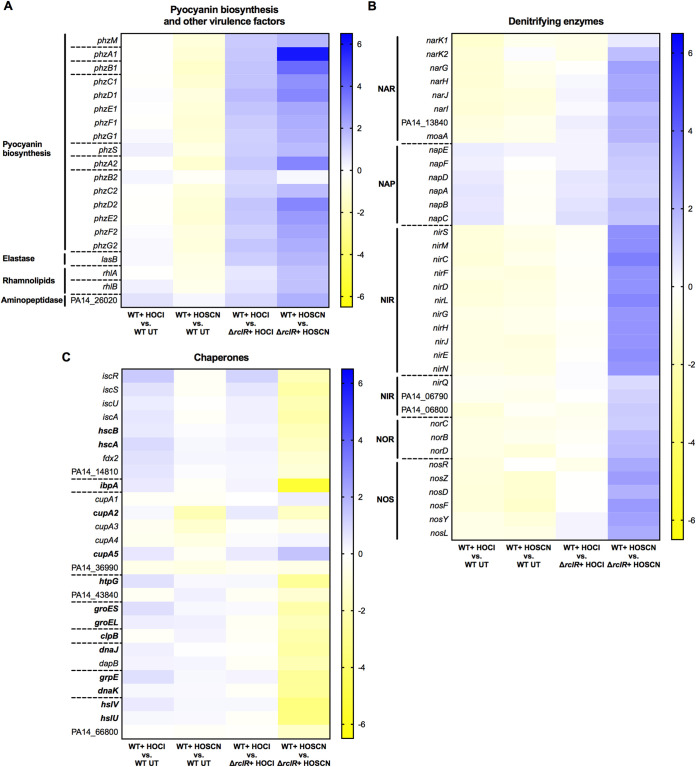
Heat maps displaying expression of pyocyanin biosynthesis, denitrifying enzyme, and chaperone and heat shock genes in PA14 WT and Δ*rclR* strains after HOCl or HOSCN exposure. (A) Pyocyanin biosynthesis and other virulence factors. (B) Denitrifying enzymes. (C) Chaperones (genes in boldface indicate the chaperones). The expression of genes is color coordinated from 6.5 log_2_ fold change (blue) to −6.5 log_2_ fold change (yellow). The first column indicates HOCl-responsive genes (log_2_ fold change of WT plus HOCl versus WT UT), and the second column indicates HOSCN-responsive genes (log_2_ fold change of WT plus HOSCN versus WT UT). The third column indicates RclR-regulated genes under HOCl stress (log_2_ fold change of WT plus HOCl versus Δ*rclR* strain plus HOCl), and the fourth column indicates RclR-regulated genes under HOSCN stress (log_2_ fold change of WT plus HOSCN versus Δ*rclR* strain plus HOSCN). In these columns, genes that are positively or negatively regulated are indicated by blue or yellow, respectively. Broken lines indicate the start and end of operons.

The expression of functionally categorized genes was displayed in heat maps to demonstrate differences in gene expression in response to HOCl (first column) or HOSCN (second column) and in which upregulated genes are displayed in blue and downregulated in yellow (Fig. 8 and 9). Genes that are positively or negatively regulated by RclR are indicated by blue or yellow, respectively, in the presence of HOCl (third column) or HOSCN (fourth column) (Fig. 8 and 9).

The genes of noncoding RNAs were upregulated in response to HOCl and HOSCN; HOCl increased the expression of tRNAs (Fig. 8A), whereas HOSCN upregulated 5S and 16S rRNAs (Data Set S1). An rRNA depletion step had been carried out prior to RNA-seq using the highly efficient Ribo-Zero kit (50), and less than 2% rRNA was recovered; therefore, the physiological importance of this induction is unclear. All of the genes that are involved in biogenesis and regulation of the T3SS (51) were induced in the WT in response to both HOCl and HOSCN, while RclR appeared to downregulate a number of the T3SS genes in the presence of HOSCN (Fig. 8B and Data Set S1). Around 40% of the genes in the transport of small molecules category that were upregulated in the WT response to HOCl and HOSCN were involved in sulfur or taurine metabolism (Fig. 8C and Fig. S9B and Data Set S1). The *mexEF-oprN* operon, which encodes the efflux pump that we found plays a role in protection against HOCl (Fig. 2A), was upregulated in response to HOCl and HOSCN exposure (Fig. 8D and Data Set S1). The *mexT* regulator did not have altered gene expression (Fig. 8D and Data Set S1). Surprisingly, these data indicated that RclR downregulates the expression of the *mexEF-oprN* operon in the presence of HOCl and HOSCN (Fig. 8D and Data Set S1). The MexT-regulated operon, *pyeRM-xenB*, which is also involved in protection against HOCl (Fig. 2C), was upregulated in response to HOCl but was downregulated in response to HOSCN, and this appeared to be dependent on RclR (Fig. 8D and Data Set S1).

### RclR regulates expression of pyocyanin biosynthesis genes in the presence of HOCl and HOSCN and denitrification and chaperone genes in the presence of HOSCN.

Heat maps of functionally categorized genes with altered expression in the Δ*rclR* deletion mutant included *phz*, denitrification, and chaperone genes. The *phz* genes are involved in the biosynthesis of the virulence factor pyocyanin (52). The overall WT response to HOCl and HOSCN had no and little effect, respectively, on *phz* expression (Fig. 9A and Data Set S1). However, the Δ*rclR* mutant displayed lower *phz* expression levels after HOCl and HOSCN exposure than the WT; thus, when values of the treated WT were divided by those for the treated Δ*rclR* strain, the results indicate that RclR positively regulates *phz* gene expression in the presence of both oxidants (Fig. 9A and Data Set S1). In the absence of these oxidants, there was no difference between pyocyanin production in WT and Δ*rclR* strains (data not shown). Similarly, the virulence factors elastase (*lasB*) and rhamnolipids (*rhlA* and *rhlB*) and an aminopeptidase (PA14_26020) did not display altered expression in response to HOCl or HOSCN in the WT but were upregulated by RclR in the presence of both oxidants (Fig. 9A and Data Set S1). In the WT strain, HOCl and HOSCN had little effect on the expression of genes required for anaerobic dissimilatory denitrification (Fig. 9B and Data Set S1). However, in the presence of HOSCN, but not HOCl, RclR positively regulated the expression of 6 operons encoding the denitrifying enzymes: nitrate reductase (NAR), periplasmic nitrate reductase (NAP), nitrite reductase (NIR), nitric oxide reductase (NOR), and nitrous oxide reductase (NOS) (Fig. 9B and Data Set S1). HOCl and HOSCN had little effect on the expression of chaperone and heat shock genes in the WT strain (Fig. 9C and Data Set S1). RclR did not regulate the expression of these genes in the presence of HOCl, yet in the presence of HOSCN, RclR negatively regulated the expression of 13 chaperone and heat shock genes, *hcsB*, *hcsA*, *ibpA*, *cupA2*, *htpG*, *groES*, *groEL*, *clpB*, *dnaJ*, *grpE*, *dnaK*, *hslV*, and *hslU*, and positively regulated the expression of the chaperone gene *cupA5* (Fig. 9C and Data Set S1).

## DISCUSSION

A major mechanism employed by the innate immune response to attack infecting bacteria, such as during lung infections, is through the production and release of the antimicrobial oxidants HOCl and HOSCN by neutrophils and the airway epithelium, respectively (7, 9).

In this study, we investigated how P. aeruginosa adapts to and protects itself against HOCl and HOSCN. This is of direct relevance to CF infections due to evidence of a compromised immune response in CF patients, including impaired HOCl and HOSCN formation (53–57), and evidence for HOSCN production being protective of lung function (58).

Our mutant screen identified regulators with known or putative roles in antibiotic resistance, methionine biosynthesis, catabolite repression, and the antioxidant response that are required for protecting P. aeruginosa against HOCl (Fig. 2, 3, and 4). We revealed that the RclR transcriptional regulator, the P. aeruginosa homologue of the E. coli RclR HOCl-specific sensor (17), is required specifically for protection of P. aeruginosa against HOCl and HOSCN and responds to the presence of these oxidants through activating expression of its adjacent gene, *rclX* (Fig. 5, 6, and 7).

E. coli RclR (RclR_Ec_) also induces the expression of its adjacent operon, *rclABC*, in the presence of HOCl (17). However, unlike *rclR_Ec_* (17), we found P. aeruginosa *rclR* (*rclR_Pa_*) is constitutively expressed, not upregulated by HOCl (or HOSCN), and does not regulate its own expression (Fig. 6A). The *rclX* gene regulated by RclR_Pa_ encodes a putative peroxiredoxin, is one of the most upregulated genes in response to HOCl and HOSCN exposure, and is required for protection against both oxidants (Fig. 5, 6, and 7; see also Fig. S4 in the supplemental material). RclX is not homologous to any of the genes in the RclR_Ec_-regulated *rclABC* operon (17), indicating differing approaches to protection against HOCl between these Gram-negative bacteria. Homology modeling and multiple-sequence alignment support RclX being an AhpD-like protein, with 4 conserved catalytic residues, including the CXXC motif (Fig. S7), which is required for peroxidase activity in the functionally characterized AhpD from M. tuberculosis (47, 48). Therefore, it is probable RclX acts as a detoxification enzyme in the protective response to HOCl and HOSCN. The sensitivity of Δ*rclR* and Δ*rclX* mutants to HOCl and HOSCN, when grown in ASM in both planktonic and biofilm cultures (Fig. S5 and S6), together with HOCl- and HOSCN-dependent expression of *rclX* in clinical CF isolates of P. aeruginosa (Fig. 6B), indicates that the RclR-mediated response is important in clinical strains and may play a role during infection.

There was considerable overlap in the transcriptional response of P. aeruginosa to HOCl and HOSCN (Fig. S9A and B). RclR regulated expression of a larger number of genes in the presence of HOSCN than HOCl (Fig. S9C and D). HOSCN caused RclR-dependent upregulation of pyocyanin biosynthesis genes and denitrification genes and downregulation of chaperone and heat shock genes (Fig. 9). Three other transcriptional regulators that are HOCl responsive in other bacteria, NemR (PA14_36300), HypT (PA14_71640), and OhrR (PA14_27230) (13, 15, 16), did not have altered expression in response to HOCl or HOSCN (Data Set S1). In a previous transcriptome study, Groitl et al. reported that P. aeruginosa *nemR* was induced by HOCl and HOSCN and P. aeruginosa *hypT* was induced by HOCl (25). Nevertheless, we demonstrate consistencies in our results, as *nemR*, *hypT*, and *ohrR* were not HOCl responsive and mutants in these genes were not HOCl sensitive (Fig. S2). The reasons for these differences between the Groitl et al. study and ours are unclear, but they may be due to variations in the growth conditions used, in particular the media used and the carbon sources metabolized.

**FIG 10:**
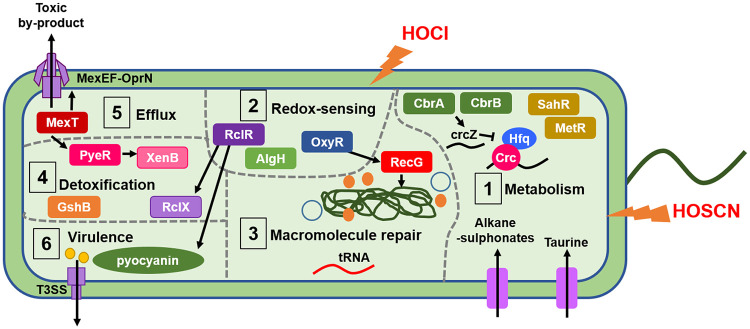
Model of the putative defense mechanisms used by P. aeruginosa in response to HOCl and HOSCN stress. In step 1, P. aeruginosa responds to HOCl and HOSCN by upregulation of sulfur transport and metabolism genes; this may facilitate increased uptake of alternative sulfur sources, including taurine, to replenish depleted sulfur levels caused by HOCl and HOSCN oxidative damage. Methionine biosynthesis and metabolism regulators MetR and SahR appear required to protect against HOCl, which we postulate is a response to oxidative disruption of the metabolism of the sulfur-containing amino acid methionine and the cellular need to maintain its levels. Catabolite repression by the Crc protein appears required for protection against HOCl, possibly indicating that optimal regulation of metabolic flexibility aids defense against HOCl. In step 2, the transcriptional regulator RclR, the H_2_O_2_ sensor OxyR (42), and the hypothetical protein AlgH (41) all have putative redox-sensing capabilities and were identified as playing a role in protection against HOCl. In step 3, macromolecular repair mechanisms are implicated in the response to HOCl and HOSCN. Upregulation of tRNAs in response to HOCl may occur to replace oxidatively damaged tRNA. The DNA repair enzyme RecG is required for protection against HOCl. In step 4, putative bacterial detoxification enzymes are involved in the response to HOCl and HOSCN. The PyeR-regulated oxidoreductase XenB is induced in response to HOCl and is required for protection, and the glutathione synthetase GshB is required for protection against HOCl. The peroxiredoxin RclX is positively regulated by RclR in response to HOCl and HOSCN and required for protection against both oxidants. In step 5, upregulation of the MexT-regulated *mexEF-oprN* operon, encoding a multidrug efflux pump (28, 29), occurs in response to HOCl and HOSCN and is required for protection against HOCl. This highlights a link between oxidative stress and antibiotic resistance and indicates a role for the efflux pump in expelling toxic by-products of these oxidants. In step 6, RclR is required for survival against HOCl and HOSCN and regulates a number of genes in the presence of both oxidants, including upregulation of the *phz* genes, which synthesize the virulence factor pyocyanin (52). The genes responsible for the biogenesis and regulation of another virulence mechanism, the T3SS (51), are upregulated in response to HOCl and HOSCN. Induction of pyocyanin and the T3SS may occur as a mechanism to counterattack the innate immune cell-derived HOCl and HOSCN-mediated oxidative attack on the bacteria.

Our study revealed that the bacterial response to HOCl and HOSCN is multifaceted and incorporates an array of genes from different systems, including metabolism, redox sensing, macromolecule repair and detoxification, export of toxic compounds, and virulence. Figure 10 summarizes the physiological processes described here as having putative roles in protecting P. aeruginosa against HOCl and HOSCN *in vitro* and, we hypothesize, in the CF lung during infection. Sulfur transporter and metabolism genes were upregulated following HOCl and HOSCN exposure, and methionine metabolism regulators SahR and MetR appear required for survival against HOCl (Fig. 3A and 8C). During sulfur starvation, bacteria are able to use alkane sulfonates and taurine as a sulfur source (59–62). Therefore, it is plausible that P. aeruginosa requires increased uptake of alternative sulfur sources and careful control of methionine metabolism to maintain levels of sulfur-containing compounds that are under oxidative attack from HOCl and HOSCN. Upregulation of sulfur transport and metabolism, and methionine and cysteine biosynthesis genes, in response to HOCl stress has been reported in other bacteria (12, 63–65). We found the catabolite repression control system (38) plays a role in protection against HOCl; this indicates that appropriate regulation of carbon metabolism is important for survival against HOCl (Fig. 3B to D). RclR upregulated the expression of denitrification genes in the presence of HOSCN (Fig. 9B). As our experiments were carried out aerobically, we would expect these Anr-regulated genes to be downregulated (66); this suggests there is a physiological advantage to expressing these pathways aerobically in the presence of HOSCN.

Genes involved in repairing macromolecules and the detoxification of oxidants were found to be important in the defense against HOCl and HOSCN. The H_2_O_2_ sensor OxyR and DNA repair enzyme RecG (42) were required for protection against HOCl (Fig. 4B). Noncoding tRNAs were upregulated in response to HOCl (Fig. 8A), perhaps to replace oxidatively damaged tRNA. Surprisingly, tRNA-Cys expression was unchanged in the presence of HOCl. RclR downregulated the expression of 13 chaperone and heat shock genes and upregulated the expression of the chaperone gene *cupA5* in the presence of HOSCN but not HOCl (Fig. 9C). In the WT strain, expression of the chaperone genes was not markedly altered in the presence of HOCl or HOSCN (Fig. 9C). In contrast, Groitl et al. found that HOCl and HOBr induced transcription of chaperone and heat shock genes and, together with HOSCN, increased production of the chaperone polyphosphate, which protects P. aeruginosa against protein unfolding and aggregation caused by the oxidants (25). As previously mentioned, these differences may be due to variations in methodological approaches used in our and the Groitl et al. studies, including the growth media and oxidant concentrations.

We found detoxification enzymes, in addition to RclX, were required for survival against HOCl, including the *pyeRM-xenB* operon, which encodes the oxidoreductase XenB (33) (Fig. 2C). The glutathione synthetase GshB was required for protection against HOCl (Fig. 4A); this enzyme is involved in synthesizing glutathione, which maintains the redox potential of the cell in response to oxidative stress (12, 67). Previous transcriptome assays have highlighted the importance of repair and detoxifying enzymes in the response of bacteria to HOCl stress (63–65, 68–71).

A striking finding of this work is a connection between the protective response to HOCl and HOSCN stress and antibiotic resistance. MexT and its regulated multidrug efflux pump, MexEF-OprN, were required for survival against HOCl (Fig. 2A). Additionally, the *mexEF-oprN* operon was upregulated in response to both HOCl and HOSCN (Fig. 8D), consistent with recent findings (25). While the fluoroquinolone-, chloramphenicol-, and trimethoprim-exporting MexEF-OprN efflux pump is normally quiescent in WT cells or not expressed in strains with an inactive chromosomal copy of *mexT*, it is highly induced in *nfxC*-type phenotypic mutants (*mexT*, *mexS*, or *mvaT*) in a MexT-dependent manner (72–73). This leads to increased resistance to fluoroquinolones and chloramphenicol and hypersensitivity to β-lactams (73). Of direct relevance to CF lung infections is the repeated observation that mutations in *mexT* occur in persistently infecting P. aeruginosa, both loss-of-function and gain-of-function mutations (*nfxC* type) (74–77).

MexT has been shown previously to be a redox-responsive regulator modulating the response to electrophilic and nitrosative stress (78, 79). Our data extend the role of MexT and MexEF-OprN to protection of P. aeruginosa against HOCl and HOSCN stress and suggest that the cell has a pleiotropic need for induction of the MexEF-OprN efflux pump in response to a wide range of oxidative stress events. MexEF-OprN can expel products other than antibiotics, and increased expression of *mexEF-oprN* is associated with decreased quorum sensing due to the efflux of quorum-sensing molecules, specifically the *Pseudomonas* quinolone signal precursors 4-hydroxy-2-heptylquinoline and kynurenine (80, 81). Therefore, one can propose that MexEF-OprN might additionally expel toxic by-products of HOCl and HOSCN reactions.

In conclusion, our findings have identified a wide range of different genes involved in the bacterial response to HOCl and HOSCN stress and provide the foundation from which further exploration of these mechanisms in protecting P. aeruginosa from these oxidants in the context of infection can be conducted.

## MATERIALS AND METHODS

### Bacterial strains, plasmids, and growth conditions.

All strains, plasmids, and primers used in this study are listed in Table S1 in the supplemental material. Three anonymized clinical isolates of P. aeruginosa from airway secretions of infected CF patients were obtained from the Royal Brompton Hospital. PCR products and plasmids were sequenced by GENEWIZ, Inc. Bacteria were routinely grown aerobically at 37°C, with shaking at 200 rpm or 700 rpm when in 96-well plates, in LB medium (5 g/liter NaCl, 5 g/liter yeast, and 10 g/liter tryptone) or on LBA plates (LB and 1.5% [wt/vol] agar) supplemented with appropriate antibiotics. P. aeruginosa strains were isolated on *Pseudomonas* isolation agar (PIA) (Sigma) supplemented with 20% (vol/vol) glycerol. ASM was prepared as described by Kirchner et al.; however, all individual amino acids, apart from l-tryptophan, were replaced with Casamino Acids, as described by Sriramulu et al. (43, 44). The components of ASM per liter were 4 g low-molecular-weight salmon sperm DNA, 5 g mucin from porcine stomach (type II), 4.75 g Casamino Acids, 0.25 g l-tryptophan, 5 g NaCl, 2.2 g KCl, 5.9 mg diethylenetriaminepentaacetic acid (DTPA), and 5 ml egg yolk emulsion. The pH of ASM was adjusted to 6.9, filter sterilized (44), and stored at 4°C for up to 1 month. After filtration, the ASM slowly turns cloudy due to precipitation of the salts from the media, but this does not impact its quality. Pyocyanin was extracted from 20-h cultures grown in LB, and the concentration was expressed in micrograms per milliliter, as described previously (82).

### Construction of plasmids and mutant strains.

Details of genes were obtained from The Pseudomonas Genome Database (27). In-frame deletions were constructed as previously described (83). Briefly, for the construction of deletion mutants of PA14 *rclR*, *rclX*, *pyeRM-xenB* operon, and *oxyR*, an ∼500-bp upstream and ∼500-bp downstream fragment of the genes was amplified by PCR using primer pairs 1F and 2R as well as 3F and 4R (Table S1). Subsequent overlap PCR was performed using primers 1F and 4R (Table S1) to fuse the upstream and downstream fragments together to form a mutator fragment that was purified and ligated into the pCR-Blunt cloning vector (Invitrogen) for sequence verification using M13F and M13R primers. The *rclR*, *rclX*, and *pyeRM-xenB* mutator fragments were cloned into the BamHI site of the pKNG101 suicide vector, and the *oxyR* mutator fragment was cloned into the XbaI site of pKNG101; all were transformed into the donor CC118λpir E. coli strain and plated onto LBA supplemented with 50 μg/ml streptomycin. The *rclR*-pKNG101, *rclX*-pKNG101, *pyeRM-xenB*-pKNG101, and *oxyR*-pKNG101 plasmids from the donor strain were introduced into the PA14 recipient strain by conjugation. The DH5α E. coli strain containing the helper plasmid pRK2013 was grown on LBA supplemented with 50 μg/ml kanamycin, and the recipient PA14 strain was grown on LBA. Colonies of the donor, helper, and recipient strains were mixed together on LBA and incubated overnight. PA14 with the *rclR*-pKNG101, *rclX*-pKNG101, *pyeRM-xenB*-pKNG101, and *oxyR*-pKNG101 plasmids integrated site specifically into the chromosome as single crossovers were isolated on PIA with 2,000 μg/ml streptomycin. Subsequently, for sucrose counterselection, isolated colonies were streaked onto LBA without NaCl and with 10% (wt/vol) sucrose and incubated at room temperature for 48 h to obtain double-crossover unmarked in-frame deletion mutants. For *oxyR* deletion, 100 U of catalase from bovine liver per ml was added to the media. This is due to Δ*oxyR* mutants being unable to survive in LB, as components within the media autoxidize to generate ∼1.2 μM H_2_O_2_ min^−1^ (84). Colonies were isolated on LBA, and deletion of *rclR*, *rclX*, *pyeRM-xenB*, and *oxyR* was confirmed by PCR and sequencing using 5F and 6R primers (Table S1). Subsequent initial overnight growth of the Δ*oxyR* strain on LBA or in LB broth included 100 U/ml catalase. Complement plasmids *rclR*-pUCP18 and *rclX*-pUCP18 were constructed by PCR amplification of the promoter region and open reading frame of PA14 *rclR* and *rclX* using primers listed in Table S1 and subsequent cloning into the HindIII/XbaI sites of the pUCP18 shuttle vector. The plasmids were transformed into DH5α E. coli and grown on LBA with 100 μg/ml ampicillin prior to sequence verification using M13F and M13R primers (Table S1) and transformation into PA14 Δ*rclR* or Δ*rclX* mutant strains. The *rclR-lacZ* and *rclX-lacZ* plasmids were constructed by PCR amplification of the upstream promoter DNA (117 bp) of *rclR* and *rclX* using primers listed in Table S1 and ligation into pCR-Blunt (Invitrogen) for sequence verification using M13F and M13R primers. The promoter DNA of *rclX* and *rclR* was cloned into the EcoRI/PstI sites of the transcriptional fusion promoter-probe vector pMP220 (containing the *lacZ* gene), transformed into DH5α E. coli, and grown on LBA with 25 μg/ml tetracycline. Plasmids were sequence verified using primer Mid-PrclR or Mid-PrclX with Mid-*lacZ* (Table S1) prior to conjugation into PA14 WT and Δ*rclR* strains. Additionally, the *rclX-lacZ* plasmid was conjugated into three clinical CF isolates.

### Preparation of HOCl and HOSCN solutions.

Sodium hypochlorite solution with 10 to 15% available chlorine (Sigma) was diluted directly into LB or ASM to prepare the final HOCl concentrations required for assays. The concentration of HOCl was determined by diluting sodium hypochlorite into 40 mM potassium phosphate buffer (pH 7.5) with 10 mM sodium hydroxide and measuring the absorbance at 292 nm (ɛ, 350 M^−1 ^cm^−1^) (25, 85). HOSCN was generated enzymatically by combining glucose, glucose oxidase (GO), potassium thiocyanate (KSCN), and LPO. GO catalyzes the reaction of glucose with O_2_ to form H_2_O_2_, which reacts with KSCN, catalyzed by LPO, to form HOSCN. Stock concentrations of the enzymes, 200 U/ml GO (Sigma) and 1,000 U/ml LPO (Sigma), were prepared in 50% (vol/vol) phosphate-buffered saline (PBS) and 50% (vol/vol) glycerol solution and stored at –20°C. Stock concentrations of 4% (wt/vol) glucose and 750 mM KSCN (8 M; Fluka) were prepared in H_2_O and stored at 4°C. Varying the amount of glucose altered the amount of HOSCN produced, and a final sublethal concentration of 0.01% glucose was selected for all assays performed in LB (apart from Fig. S4, for which a final amount of 0.02% glucose was used). The final concentrations of LPO, GO, and KSCN were based on those used in reference 86. For the assays, all components were diluted directly into LB or ASM to final concentrations of 0.01% glucose (0.02% glucose for Fig. S4), 0.5 U/ml GO, 0.75 mM KSCN, and 3 U/ml LPO. A control assay without LPO that would only result in H_2_O_2_ generation (0.01% glucose, 0.5 U/ml GO, and 0.75 mM KSCN) was performed to examine the effect of H_2_O_2_ (data not shown). The concentration of HOSCN was determined by reaction with 5-thio-2-nitrobenzoic acid (TNB), as described previously (25, 87). Briefly, 10 mM 5,5′-dithiobis (2-nitrobenzoic acid) (Thermo Scientific), prepared in 40 mM potassium phosphate buffer (pH 7.5), was hydrolyzed by the addition of sodium hydroxide to generate TNB. The TNB concentration was determined by measuring absorbance at 412 nm (ɛ, 14,150 M^−1 ^cm^−1^). HOSCN (0.01% glucose [0.02% glucose for Fig. S4], 0.5 U/ml GO, 0.75 mM KSCN, and 3 U/ml LPO) prepared in 40 mM potassium phosphate buffer (pH 7.5) was diluted 1:1 in TNB, and the loss of absorption at 412 nm was measured after 20 min. The concentration of HOSCN was determined from the concentration of TNB consumed during the reaction (stoichiometry, 2:1 [TNB:HOSCN]). A concentration of 0.8 mM HOSCN was produced from 0.01% glucose and 1 mM HOSCN from 0.02% glucose. For the ASM assays, 0.8 mM HOSCN was produced, as described above, which was then diluted in ASM to give the final HOSCN concentrations used.

### HOCl and HOSCN susceptibility and viability assays.

A total of 707 strains with mutations in genes with regulatory functions were selected from the PA14 nonredundant single-transposon mutant library (26) (Table S1). The strains were grown overnight in LB medium with 15 μg/ml gentamicin, alongside a PA14 WT control grown in LB medium, in 2.2 ml 96-well deep-well plates (VWR). For the HOCl screen, overnight cultures were subcultured 1:20 into LB medium with 15 μg/ml gentamicin in 96-well microtiter plates (Falcon) and grown for 3 h. Cultures were subcultured again at 1:20 (optical density [OD], 0.05 ± 0.03) in LB medium or LB medium containing 4.4 mM HOCl in 96-well microtiter plates (Falcon) and grown for up to 24 h, with the OD at 600 nm (OD_600_) recorded as a measure of growth at hourly time points. LB medium was used for HOCl screening to facilitate growth of the large number of mutant strains, some of which may require nutrient-rich broth. The concentration of HOCl used was selected from a preliminary experiment, which identified 4.4 mM HOCl as being sublethal to PA14 WT, causing a lag in growth but not complete inhibition (data not shown). However, for the assays that tested the HOCl susceptibility of Δ*pyeRM-xenB*, 5.1 mM HOCl was used. For PA14 WT and Δ*rclR* and Δ*rclX* mutant strains, and Δ*rclR* plus *rclR*-pUCP18 and Δ*rclX* plus *rclX*-pUCP18 complemented strains, overnight cultures were prepared by growing the strains in LB medium or LB medium with 500 μg/ml carbenicillin for the pUCP18 complemented strains. Overnight cultures were subcultured 1:20 in LB medium and grown for 3 h prior to subculturing again at 1:20 (OD, 0.05 ± 0.02) into LB medium or LB medium with 4.4 mM HOCl or 0.8 mM HOSCN. We monitored growth by recording the OD_600_. The concentration of HOSCN was chosen from initial growth curve analyses, which identified 0.8 mM and 1 mM HOSCN as subinhibitory for Δ*rclR* and Δ*rclX* strains, respectively (data not shown). However, as the higher concentration was lethal to the Δ*rclR* strain, the lower concentration of 0.8 mM was used for all experiments performed in LB, apart from Fig. S4. For the HOCl and HOSCN susceptibility assays in ASM, the PA14 WT and Δ*rclR* and Δ*rclX* mutant strains were grown as described above. The only difference was the ASM was filtered again prior to adding HOCl at a final concentration of 3.1 mM and adding components to make 0.8 mM HOSCN, which was diluted to a final concentration of 0.53 mM. Lower concentrations were required in ASM, as bacteria had increased susceptibility to both oxidants in this medium. For HOCl and HOSCN viability assays, cultures were sampled at 0 h and 2.5 h or 4.5 h after HOSCN or HOCl exposure, respectively, 10-fold serial dilutions in PBS were prepared, and dilutions were drop plated on LBA. For other chemical susceptibility assays, PA14 WT, Δ*rclR*, and Δ*rclX* strains were grown as described above but in LB medium containing 0.125 mM NEM, 5 mM diamide, 2.5 mM methylglyoxal, 5 mM H_2_O_2_, 0.5 mM TBH, 1 mM methyl viologen, or 10 mM DEANO. The concentrations of chemicals used were determined from preliminary experiments that tested a range of concentrations to identify those that caused a lag, but not inhibition, in the growth of PA14 WT (data not shown). All chemicals were from Sigma, apart from TBH, which was from EMD Millipore. For ASM biofilm assays, we adapted the method from reference 44. Overnight cultures of PA14 WT, Δ*rclR*, and Δ*rclX* strains were subcultured 1:50 in LB and grown for 3 h prior to subculturing again at 1:50 into 2 ml ASM without or with HOCl (3.1 or 3.5 mM) and HOSCN (0.43 mM or 0.53 mM) in a 24-well tissue culture-treated plate (Falcon), which was incubated at 37°C, with low-speed shaking, for 3 days to allow biofilm formation and then left at room temperature, static, for a further 4 days before visualization.

### RNA extraction and RNA-seq analysis.

Exponential-phase PA14 WT and Δ*rclR* cultures were subcultured 1:50 in LB (OD, 0.05 ± 0.02) and grown for 2.5 h prior to adding HOCl to a final concentration of 2.2 mM, HOSCN to a final concentration of 0.8 mM, or no treatment (LB only). LB medium was used for consistency with the HOCl screen. The concentrations of oxidants used were chosen due to causing a 30- to 120-min lag in growth of exponential-phase cultures. Treated and untreated WT and Δ*rclR* cultures were incubated for a further 20 min, and then cells were collected and centrifuged at 13,000 × *g* for 5 min. The pellets were washed twice with PBS, and RNA*later* (Ambion) was added to preserve the RNA. Two biological replicates of WT and Δ*rclR* strains from independent experiments, treated with HOCl or HOSCN or left untreated, were collected for RNA purification. Enzymatic lysis and proteinase K digestion of bacteria followed by RNA purification were carried out using the RNeasy Protect Bacteria minikit (Qiagen). DNase treatment of RNA samples was performed using the TURBO DNA-free kit (Ambion). Removal of rRNA, preparation of cDNA libraries, and sequencing using the Illumina NextSeq 500 system and a 75-bp read length were performed by Vertis Biotechnologie AG. Reads were aligned to the P. aeruginosa UCBPP-PA14 complete genome (NCBI accession number NC_008463.1) using bowtie2, and expression values in reads per kilobase per million mapped reads (RPKM) were calculated using FeatureCounts. Normalization of the RPKM values against the OD_600_ of the samples prior to RNA extraction was performed, and the values of the replicates were averaged. The log_2_ fold change of the RPKM values of treated WT compared to untreated WT or treated WT compared to treated Δ*rclR* strain was calculated. Genes with a log_2_ fold change of >1.5 and <−1.5 were considered differentially expressed.

### Quantitative real-time PCR.

Cultures of PA14 WT were grown to mid-exponential phase in LB and treated with HOCl or HOSCN for 20 min prior to RNA extraction; this was performed as described for RNA-seq. The cDNA library was prepared using the high-capacity cDNA reverse transcription kit (Applied Biosystems). Quantitative PCR was set up with Fast SYBR green mix (Invitrogen) and using the Applied Biosystems 7500 fast real-time PCR instrument. Expression ratios were calculated by comparing the expression of each gene in treated PA14 WT cultures to untreated PA14 WT cultures by the ΔΔ*C_T_* method (88) and normalized to expression of *rpoD* (RNA polymerase sigma factor), which shows unchanged expression levels under the conditions tested. Primers used for qRT-PCR are listed in Table S1.

### β-Galactosidase assays.

PA14 WT and Δ*rclR* strains containing *rclR-lacZ* and *rclX-lacZ* plasmids, and clinical CF isolates 1, 2, and 3 containing the *rclX-lacZ* plasmid, were grown in LB medium with 100 μg/ml tetracycline overnight. Cultures were subcultured 1:50 (OD, 0.05 ± 0.02) in LB medium in 2.2-ml 96-well deep-well plates (VWR) and grown to exponential phase (3 h) and then were left untreated or treated with 2.2 mM HOCl or 0.8 mM HOSCN. Cultures were collected 30 min after treatment with HOCl or HOSCN. β-Galactosidase activity was assayed using the modified version (89) of the Miller method (90).

### Homology modeling and sequence analysis.

The predicted structure of RclX was modeled with SWISS-MODEL (91), and illustrations of protein structures were prepared with EzMol (92). Amino acid sequences were aligned using Clustal Omega (93) and visualized with ESPript 3.0 (94).

### Data availability.

The transcriptomic data of our RNA-seq experiments have been deposited in the NCBI’s GEO database (95) under accession number GSE124385.

## Supplementary Material

Supplemental file 1

Supplemental file 2
