# Supplementary material for: Response of Pseudomonas aeruginosa to the Innate Immune System-Derived Oxidants Hypochlorous Acid and Hypothiocyanous Acid
Source: J Bacteriol. 2020 Dec 18;203(2):e00300-20. doi: 10.1128/JB.00300-20 (PMC7950407; doi:10.1128/JB.00300-20)
Supplement: Supplemental file 1 [file JB.00300-20-s0001.pdf]

Supplemental information

Response of *Pseudomonas aeruginosa* to the innate immune system-derived oxidants hypochlorous acid and hypothiocyanous acid

Katie V. Farrant, Livia Spiga, Jane C. Davies and Huw D. Williams

In this document: Fig S1-S9, Table S1, Table S2
In separate excel file: Data set S1 (RNA_{seq} transcriptomic data)

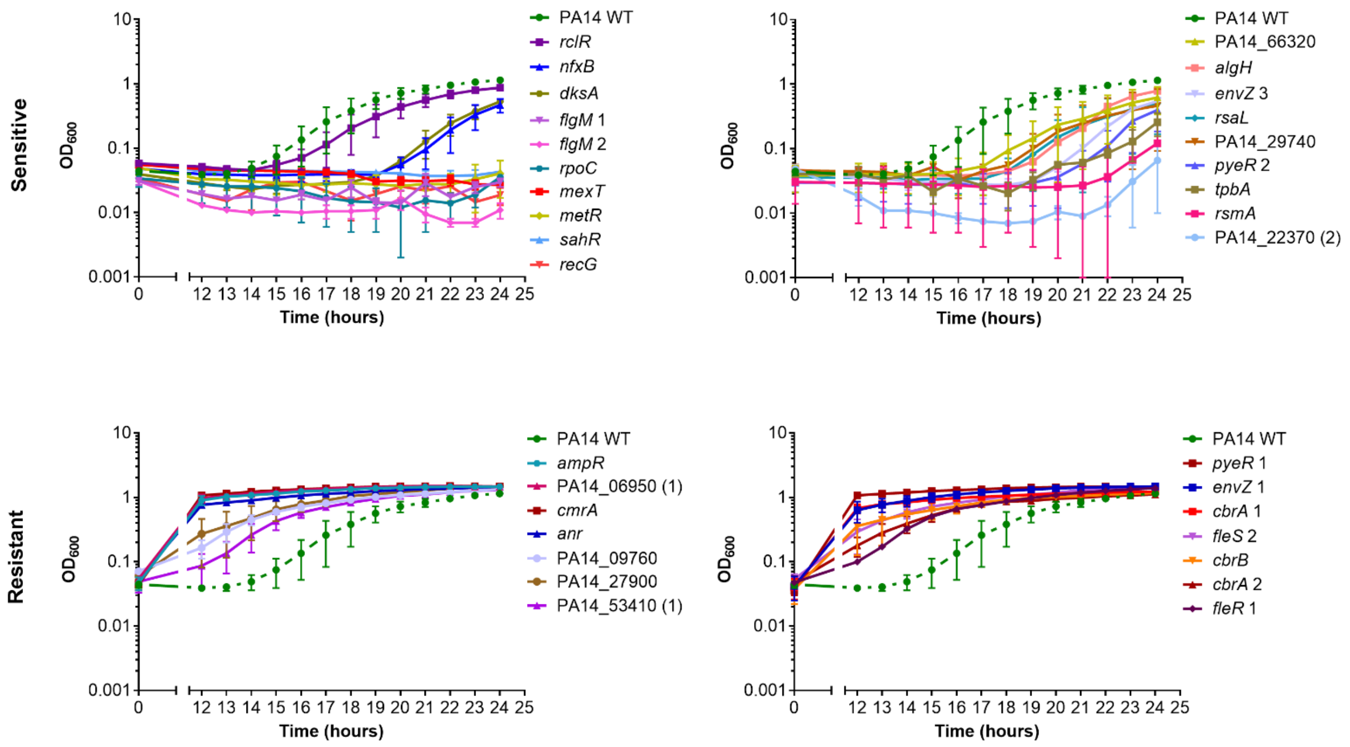

FIG S1 Rescreen of resistant and sensitive strains identified from the HOCl screen. Strains were grown in LB medium with 4.4 mM HOCl, at 37◦C with shaking. Graphs show regulatory gene mutant strains that displayed consistent HOCl-sensitive or resistant phenotypes but were not all statistically significant. The *pyeR* 1 and *envZ* 1 strains from the PA14 transposon library were constructed within the *exoU* mutant background, rather than the WT background (Table S1). We cannot rule out that this contributes to the observed opposing phenotype of *pyeR* 1 and *pyeR* 2, and *envZ* 1 and *envZ* 3. Graphs display the means and standard error of the means for two biological replicates from independent experiments. Strains grown in the absence of HOCl showed no growth defects (data not shown).

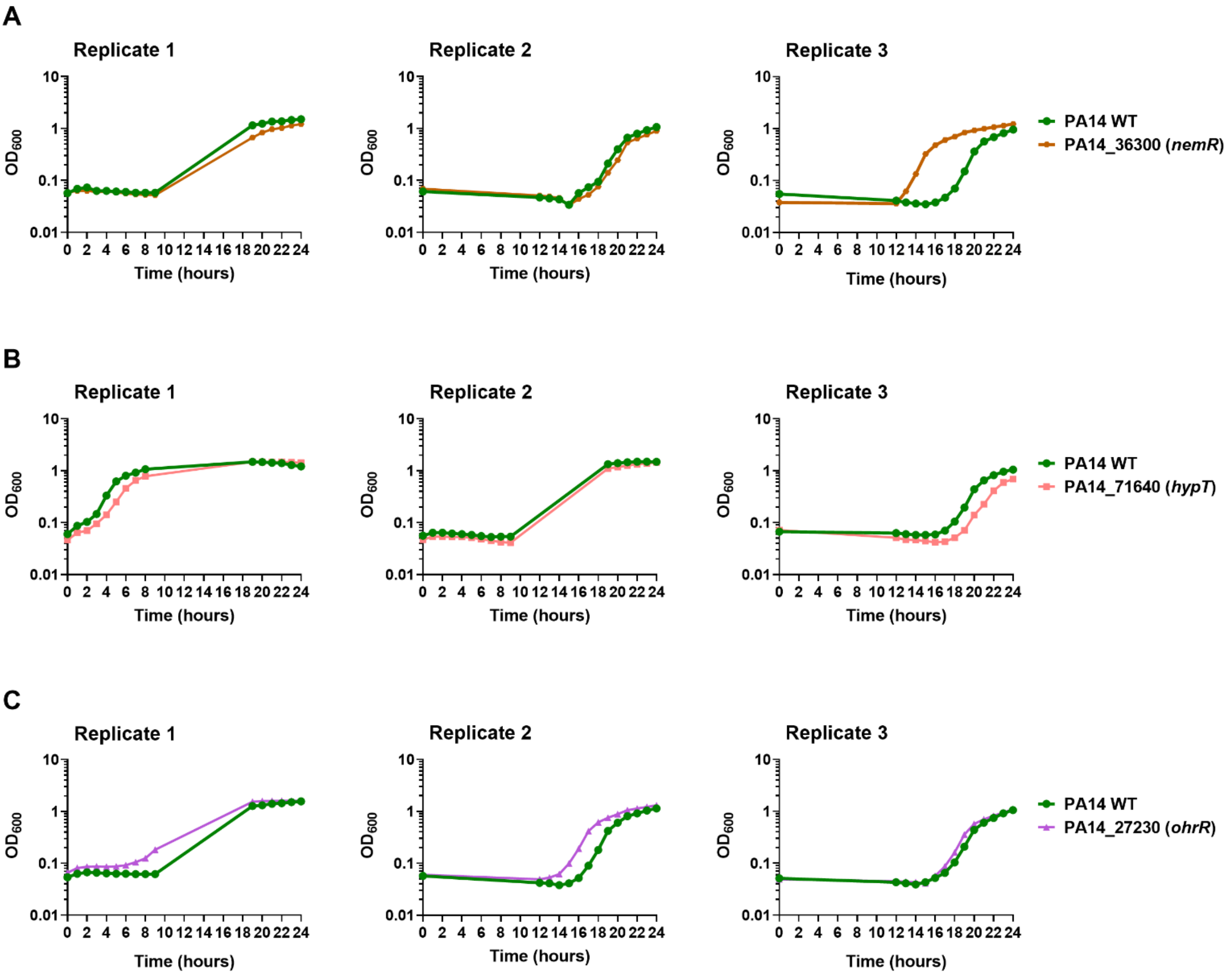

FIG S2 Mutants of genes homologous to other HOCl-responsive transcriptional regulators from *E. coli* and *B. subtilis*: *nemR*, *hypT* and *ohrR*, did not display statistically different susceptibility to HOCl. Growth of (A) PA14 WT and PA14_36300 (*nemR*) strains, (B) PA14 WT and PA14_71640 (*hypT*) strains and (C) PA14 WT and PA14_27230 (*ohrR*) strains, in the presence of 4.4 mM HOCl. Graphs show three biological replicates from independent experiments. Strains grown in the absence of HOCl showed no growth defects (data not shown).

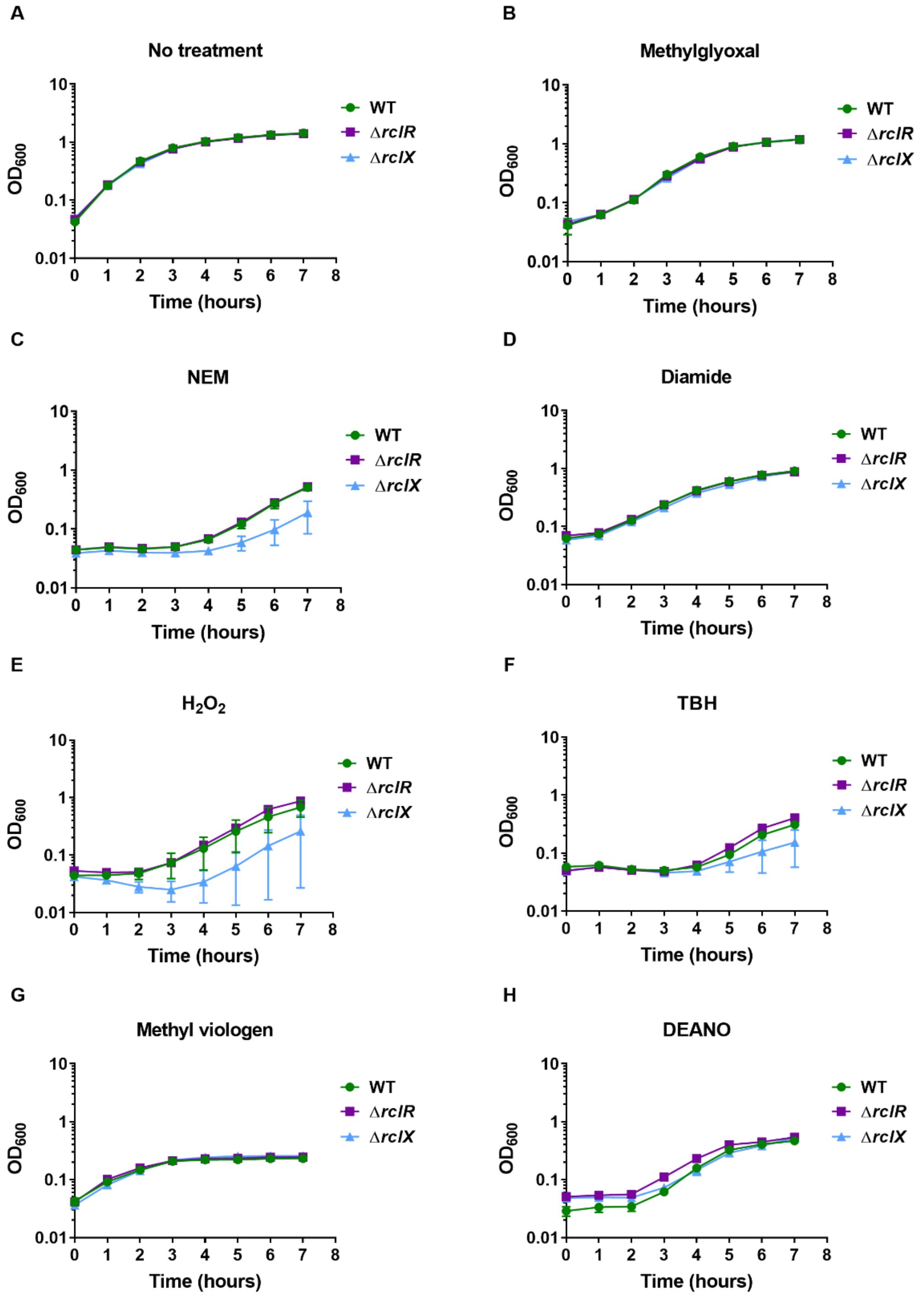

FIG S3 Susceptibility of Δ*rclR* and Δ*rclX* to various reactive oxygen, electrophilic and nitrogen species. Growth curves of PA14 WT, Δ*rclR* and Δ*rclX* in (A) LB medium, (B) LB medium with 2.5 mM methylglyoxal, (C) LB medium with 0.125 mM NEM, (D) LB medium with 5 mM diamide, (E) LB medium with 5 mM H_2O_2, (F) LB medium with 0.5 mM TBH, (G) LB medium with 1 mM methyl viologen, (H) LB medium with 10 mM DEANO. Graphs display the means and standard error of the means for (A+H) three biological replicates and (H) six biological replicates.

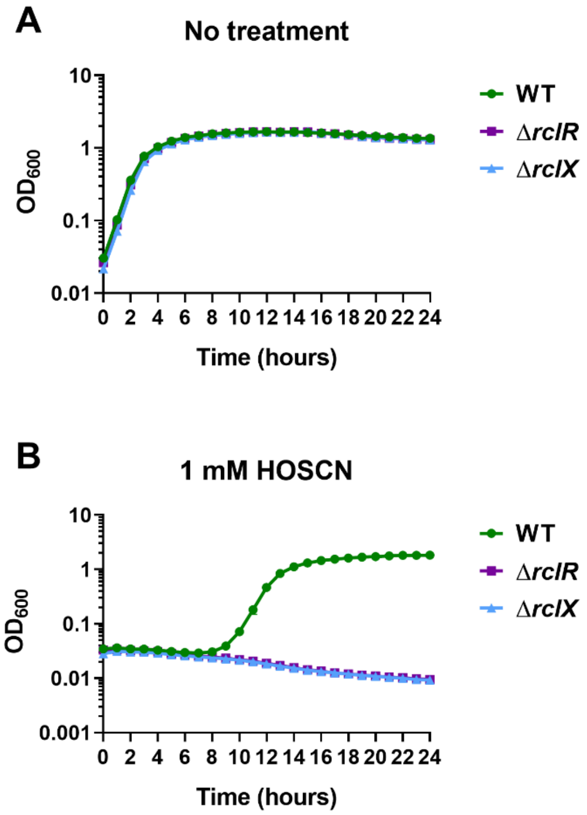

FIG S4 Both Δ*rclR* and Δ*rclX* are sensitive to a higher concentration of HOSCN. Growth curves of PA14 WT, Δ*rclR* and Δ*rclX* in (A) LB medium, and (B) LB medium with 1mM HOSCN (0.02% glucose). Graphs display the means and standard error of the means for three biological replicates, representative of at least three independent experiments.

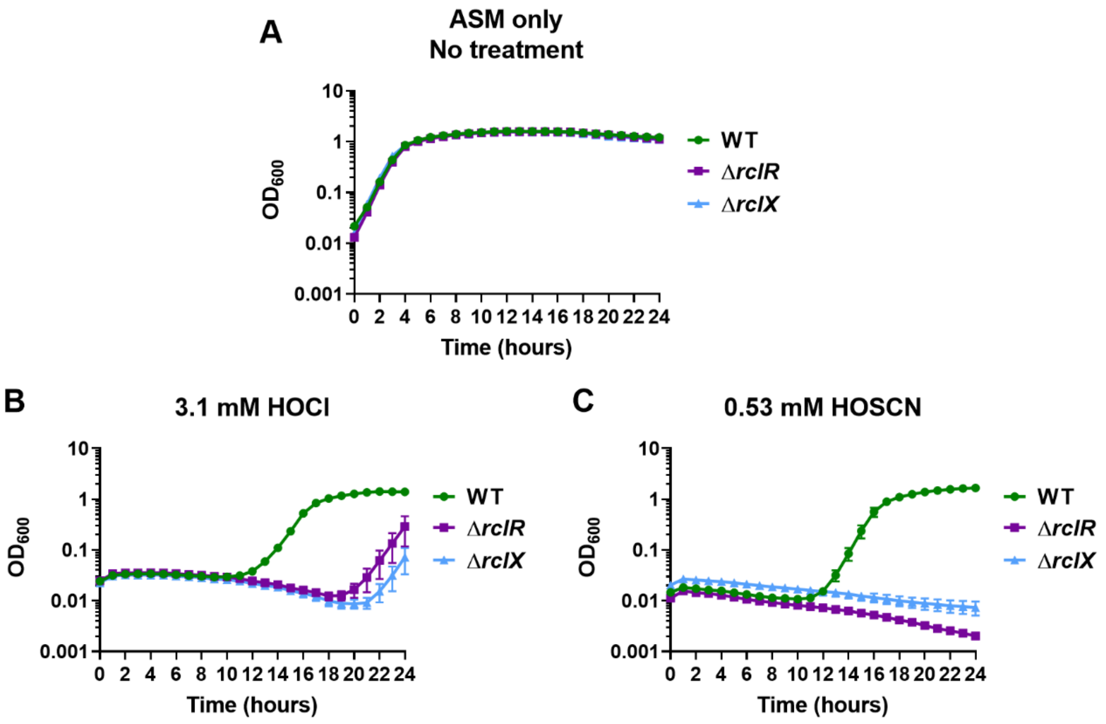

FIG S5 Both Δ*rclR* and Δ*rclX* strains are sensitive to HOCl and HOSCN when grown in ASM medium. Growth of WT, Δ*rclR* and Δ*rclX* strains in 96-well plates, at 37◦C with shaking in (A) ASM medium (no treatment), (B) ASM medium with 3.1 mM HOCl, and (C) ASM medium with 0.53 mM HOSCN. Graphs display the means and standard error of the means for three biological replicates, representative of three independent experiments.

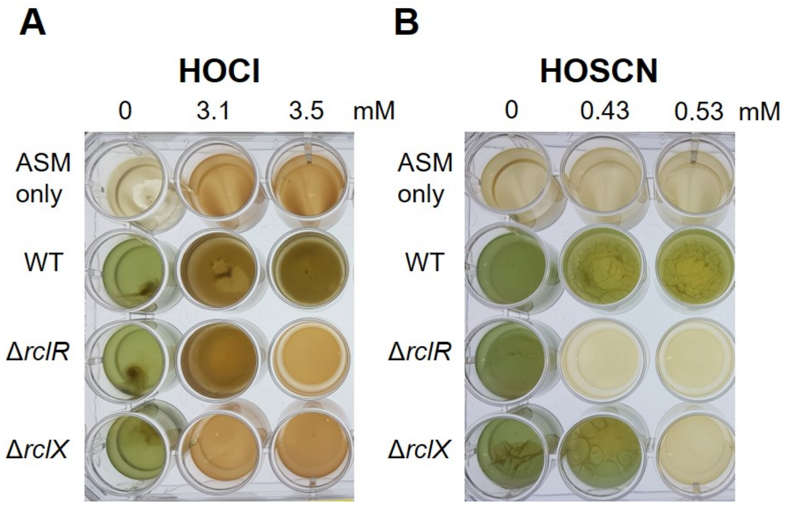

FIG S6 Mutants of *rclR* and *rclX* are sensitive to HOCl and HOSCN when grown in an ASM biofilm model. Growth of PA14 WT, Δ*rclR* and Δ*rclX* in 24-well plates in (A) ASM in the absence or presence of 3.1 and 3.5 mM HOCl or (B) ASM in the absence or presence of 0.43 mM and 0.53 mM HOSCN. Strains were grown for 3 days at 37 °C, to allow biofilms to form, before leaving at room temperature for a further 4 days when biofilms could be clearly distinguished. *P. aeruginosa* formed both visible suspended biofilms and non-visible surface attached biofilms. Images are representative of three independent experiments.

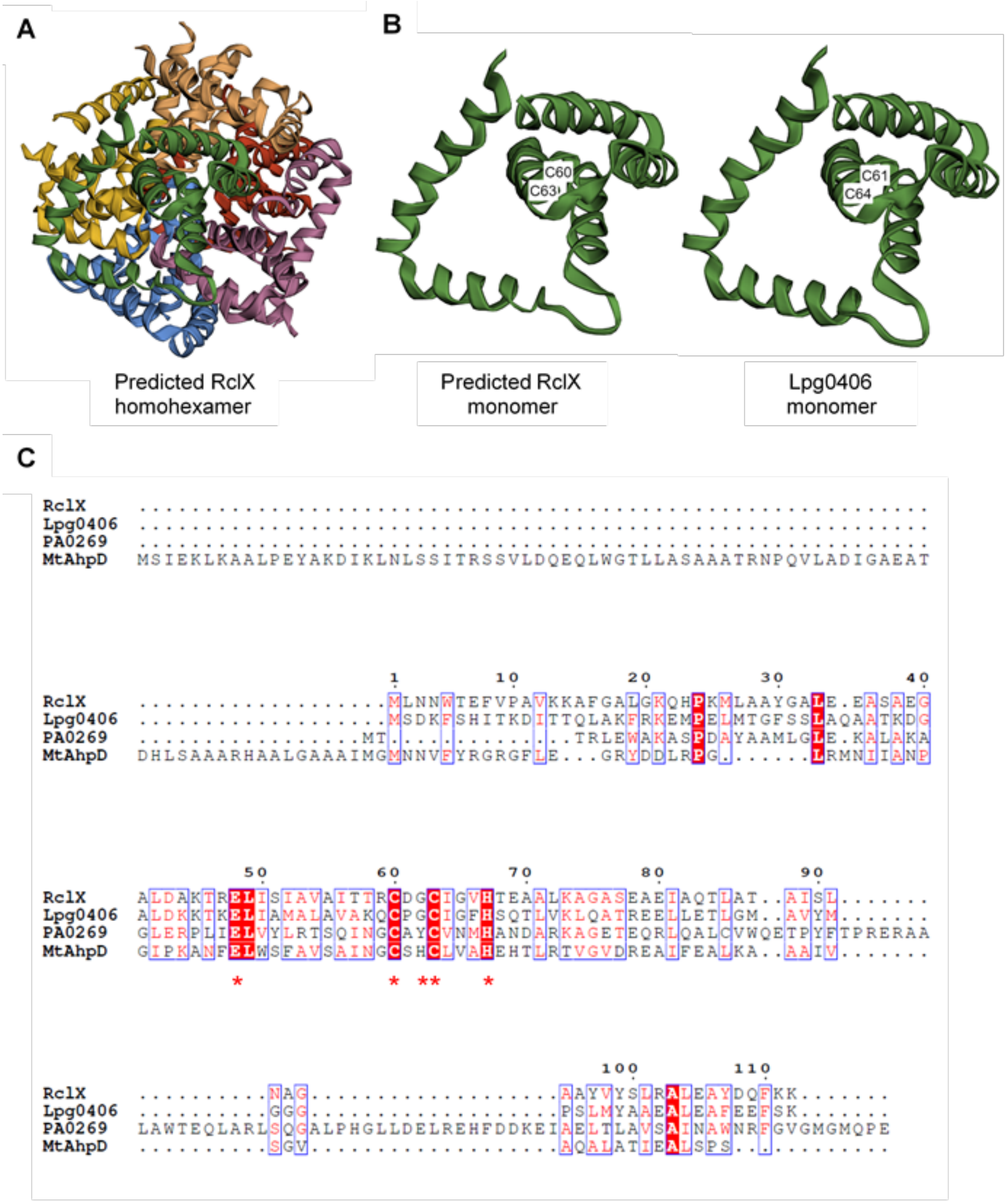

FIG S7 Predicted structure of RclX and sequence alignment with three other AhpD proteins. Ribbon representation of RclX structure based on homology modelling with Lpg0406 (PDB: 5dik) from *Legionella pneumophila*, 32.1% identical: (A) Predicted homohexamer of RclR, each monomer coloured differently, (B) Predicted monomer of RclX (left) and monomer of Lpg0406 (right) consisting of six α- helices, conserved catalytic cysteine residues are labelled. (C) Sequence alignment of RclX with Lpg0406, PA0269 from *P. aeruginosa* (15.3% identical), and MtAhpD from *Mycobacterium tuberculosis* (17% identical). Asterisks mark the five functional residues, E118, C130, H132, C133, H137, which form the proton relay system of MtAhpD (1, 2).

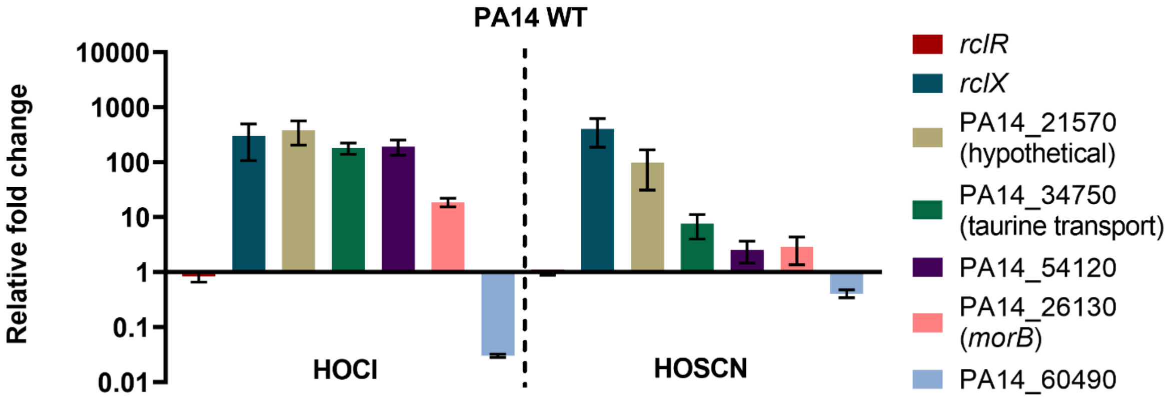

FIG S8 Altered expression of select genes in PA14 WT in response to HOCl or HOSCN exposure as determined by qRT-PCR. Relative fold changes of *rclR*, *rclX*, PA14_21570, PA14_34750, PA14_54120, PA14_26130 and PA14_60490 in PA14 WT following exposure to HOCl or HOSCN. Gene expression was normalised to the reference gene *rpoD*, which shows unchanged expression levels under the conditions tested, and fold changes were calculated relative to expression of each gene in PA14 WT untreated by the ΔΔCT method. Graphs display the means and standard error of the means for three independent experiments.

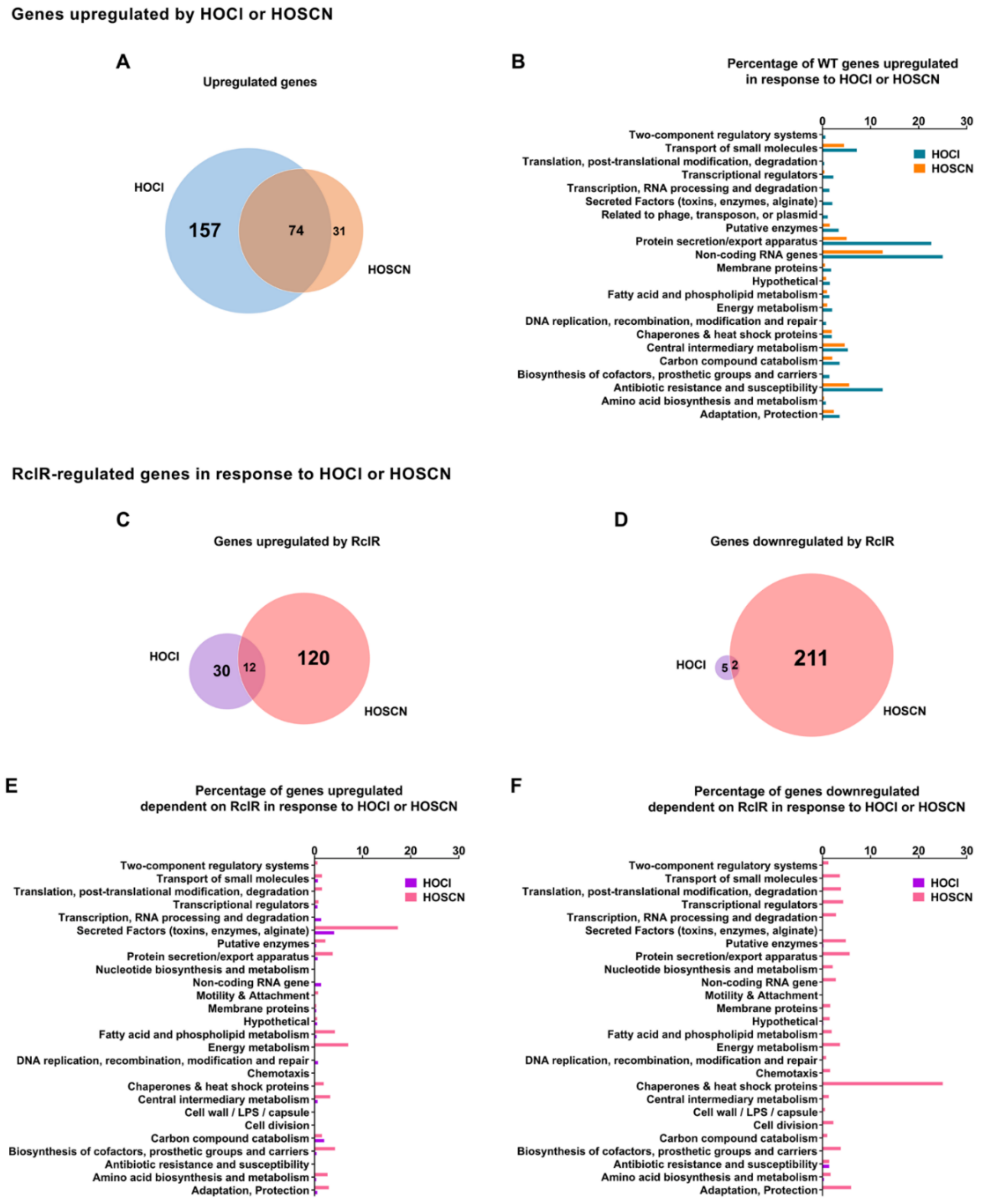

FIG S9 Numbers of upregulated or downregulated genes and functional categorisation of genes with altered expression in PA14 WT and Δ*rclR* after HOCl or HOSCN exposure. (A+B) Altered gene expression in the PA14 WT strain: (A) venn diagram of number of genes upregulated when comparing WT+ HOCl or HOSCN with WT untreated, (B) chart displays percentage of upregulated genes in different functional groups. (C-F) Altered gene expression in the Δ*rclR* strain: venn diagrams of number of genes (C) upregulated or (D) downregulated when comparing WT+ HOCl or HOSCN with Δ*rclR*+ HOCl or HOSCN and charts display percentage of (E) upregulated or (F) downregulated genes in different functional groups. Venn diagrams display number of genes >1.5 log2 fold change upregulated or <1.5 log2 fold change downregulated. Charts display genes categorised into functional groups based on assigned gene ontologies in the *Pseudomonas* genome library and expressed as a percentage of the entire functional category in the PA14 genome.

TABLE S1 Strains, plasmids and primers used in this study.

**Strain**	**Relevant characteristics**	**Source/Library plate position**
Pseudomonas aeruginosa		
PA14	Wild-type *P. aeruginosa*, clinical isolate CF lung	M. Ausubel, Harvard (3)
PA14 transposon insertion mutant library ***707 regulatory mutants***	**ExMr: ExoUpsi3 background, MAR2xT7 transposon, Gm^r** **PAMr: PA14 wild-type background, MAR2xT7 transposon, Gm^r**	**M. Ausubel, Harvard** (4)
PA14_00340	Sigma factor	PAMr_nr_mas_02_3 F2
PA14_00400	Transcriptional regulator	PAMr_nr_mas_01_3 D1
PA14_00430	Two-component system	PAMr_nr_mas_12_3 D3
PA14_00440 (*trpA*)	Two-component system	PAMr_nr_mas_07_2 A2
PA14_00450 (*trpB*)	Two-component system	PAMr_nr_mas_10_4 F9
PA14_00460 (*trpI*)	Transcriptional regulator	PAMr_nr_mas_04_1 C9
PA14_00600	Transcriptional regulator	PAMr_nr_mas_06_2 D9
PA14_00680	Transcriptional regulator	ExMr_nr_mas_01_2 E1
PA14_00875 (*ppkA*)	Serine/Threonine kinase	PAMr_nr_mas_01_1 H3
PA14_01410	Transcriptional regulator	PAMr_nr_mas_10_4 E5
PA14_01460	Two-component system	PAMr_nr_mas_08_2 F10
PA14_01470 (1)	Transcriptional regulator	PAMr_nr_mas_06_3 C8
PA14_01470 (2)	Transcriptional regulator	PAMr_nr_mas_13_1 C6
PA14_01480	Transcriptional regulator	PAMr_nr_mas_02_3 A5
PA14_01500	Transcriptional regulator	PAMr_nr_mas_07_1 A10
PA14_01640	Transcriptional regulator	PAMr_nr_mas_01_1 A9
PA14_01840	Sigma factor	PAMr_nr_mas_02_3 F11
PA14_01860	Transmembrane sensor	PAMr_nr_mas_09_4 B12
PA14_01980	Transcriptional regulator	PAMr_nr_mas_08_4 D7
PA14_02030	Transcriptional regulator	ExMr_nr_mas_01_2 E9
PA14_02090	Transcriptional regulator	PAMr_nr_mas_07_4 A5
PA14_02110	Two-component system	PAMr_nr_mas_13_4 F9
PA14_02230 (*cheW*)	Two-component system	PAMr_nr_mas_02_3 A11
PA14_02250 (*cheA*) (1)	Two-component system	PAMr_nr_mas_03_3 A1
PA14_02250 (*cheA*) (2)	Two-component system	PAMr_nr_mas_03_3 A3
PA14_02260	Two-component system	PAMr_nr_mas_03_2 G8
PA14_02290	Transcriptional regulator	PAMr_nr_mas_10_4 G8
PA14_02660 (1)	Transcriptional regulator	PAMr_nr_mas_10_2 G9
PA14_02660 (2)	Transcriptional regulator	PAMr_nr_mas_15_1 C8
PA14_02750	Transcriptional regulator	PAMr_nr_mas_01_4 E4
PA14_02910	Transcriptional regulator	PAMr_nr_mas_05_2 A4
PA14_03010	Transcriptional regulator	PAMr_nr_mas_12_4 H1

PA14_03070	Transcriptional regulator	PAMr_nr_mas_04_3 E8
PA14_03480	Transcriptional regulator	PAMr_nr_mas_04_4 A8
PA14_03530	Transcriptional regulator	PAMr_nr_mas_02_2 G3
PA14_03580	Transcriptional regulator	PAMr_nr_mas_13_2 G4
PA14_03720	Two-component system	PAMr_nr_mas_04_3 D7
PA14_03780	Transcriptional regulator	PAMr_nr_mas_12_1 H8
PA14_03790	Two-component system	PAMr_nr_mas_10_1 F12
PA14_03840 (*aguR*)	Transcriptional regulator	PAMr_nr_mas_08_3 F1
PA14_03860	Two-component system	PAMr_nr_mas_12_3 A5
PA14_03880 (*spuB*)	Two-component system	PAMr_nr_mas_07_1 D2
PA14_04000 (1)	Transcriptional regulator	PAMr_nr_mas_07_2 G4
PA14_04000 (2)	Transcriptional regulator	PAMr_nr_mas_10_4 D5
PA14_04160	Transcriptional regulator	PAMr_nr_mas_03_2 D6
PA14_04270	Transcriptional regulator	PAMr_nr_mas_10_2 D6
PA14_04420	Two-component system	PAMr_nr_mas_14_1 A10
PA14_04660	Two-component system	PAMr_nr_mas_13_4 E3
PA14_04820	Transcriptional regulator	PAMr_nr_mas_02_3 H3
PA14_05290 (*algH*)	Transcriptional regulator	PAMr_nr_mas_07_3 H10
PA14_05320 (*pilG*)	Two-component system	PAMr_nr_mas_02_4 F3
PA14_05330 (*pilH*)	Two-component system	PAMr_nr_mas_11_3 D1
PA14_05340 (*pilI*)	Two-component system	PAMr_nr_mas_11_3 G8
PA14_05360 (*pilJ*) (1)	Two-component system	PAMr_nr_mas_08_2 D11
PA14_05360 (*pilJ*) (2)	Two-component system	PAMr_nr_mas_12_2 E9
PA14_05380 (*pilK*)	Two-component system	PAMr_nr_mas_12_4 E5
PA14_05390 (*chpA*)	Two-component system	PAMr_nr_mas_07_1 G8
PA14_05410	Two-component system	PAMr_nr_mas_03_3 G9
PA14_05420	Transcriptional regulator	PAMr_nr_mas_11_1 A9
PA14_05660	Transcriptional regulator	PAMr_nr_mas_05_4 G8
PA14_05850	Transcriptional regulator	PAMr_nr_mas_08_4 E1
PA14_06060 (*creB*)	Two-component system	PAMr_nr_mas_09_2 C6
PA14_06070 (*creC*)	Two-component system	PAMr_nr_mas_02_4 D10
PA14_06180	Sigma factor	PAMr_nr_mas_02_2 B5
PA14_06210	Transcriptional regulator	PAMr_nr_mas_04_1 H7
PA14_06240	Transcriptional regulator	PAMr_nr_mas_09_2 A11
PA14_06260	Transcriptional regulator	PAMr_nr_mas_02_4 C11
PA14_06310	Two-component system	PAMr_nr_mas_06_2 C12
PA14_06330	Serine/Threonine kinase	PAMr_nr_mas_10_4 H6
PA14_06340	Molybdenum transport regulator	PAMr_nr_mas_10_1 C4
PA14_06400 (1)	Transcriptional regulator	PAMr_nr_mas_10_1 H11
PA14_06400 (2)	Transcriptional regulator	PAMr_nr_mas_10_2 C9
PA14_06690 (*nirG*)	Transcriptional regulator	PAMr_nr_mas_07_2 G1
PA14_06710	Transcriptional regulator	PAMr_nr_mas_13_1 D9

PA14_06770 (*nirQ*) (1)	Nucleoside triphosphate hydrolase	PAMr_nr_mas_01_1 E6
PA14_06770 (*nirQ*) (2)	Nucleoside triphosphate hydrolase	PAMr_nr_mas_12_4 G10
PA14_06870 (*dnr*)	Transcriptional regulator	PAMr_nr_mas_06_1 E7
PA14_06880	Transcriptional regulator	PAMr_nr_mas_12_1 D7
PA14_06950 (1)	Transcriptional regulator	ExMr_nr_mas_01_1 C10
PA14_06950 (2)	Transcriptional regulator	PAMr_nr_mas_03_1 B8
PA14_07110 (*sahR*)	Transcriptional regulator	PAMr_nr_mas_14_1 E11
PA14_07340 (*rclR*)	Transcriptional regulator	PAMr_nr_mas_03_2 D2
PA14_07680	Two-component system	PAMr_nr_mas_02_3 F8
PA14_07780	Serine/Threonine kinase	PAMr_nr_mas_03_4 B4
PA14_07820	Two-component system	PAMr_nr_mas_08_3 E1
PA14_07840	Two-component system	PAMr_nr_mas_04_3 D10
PA14_07940 (*trpE*) (1)	Two-component system	PAMr_nr_mas_09_3 F8
PA14_07940 (*trpE*) (2)	Two-component system	PAMr_nr_mas_11_4 B8
PA14_07950 (*prtN*)	Transcriptional regulator	PAMr_nr_mas_12_2 H4
PA14_08350 (*trpD*)	Two-component system	PAMr_nr_mas_09_1 E3
PA14_08360 (*trpC*)	Two-component system	PAMr_nr_mas_12_3 G6
PA14_08370 (*vfr*)	Transcriptional regulator	PAMr_nr_mas_12_4 A4
PA14_08780 (*rpoC*)	Sigma factor	PAMr_nr_mas_10_2 G3
PA14_09150 (*katA*) (1)	Catalase	PAMr_nr_mas_04_2 H3
PA14_09150 (*katA*) (2)	Catalase	PAMr_nr_mas_04_4 D2
PA14_09150 (*katA*) (3)	Catalase	PAMr_nr_mas_15_3 A3
PA14_09260 (*pchR*)	Transcriptional regulator	PAMr_nr_mas_07_4 B6
PA14_09570	Transcriptional regulator	PAMr_nr_mas_05_3 E1
PA14_09680 (1)	Two-component system	PAMr_nr_mas_11_3 E10
PA14_09680 (2)	Two-component system	PAMr_nr_mas_13_2 A10
PA14_09690	Two-component system	PAMr_nr_mas_03_4 F6
PA14_09760	Transcriptional regulator	ExMr_nr_mas_01_2 H4
PA14_09770	Transcriptional regulator	PAMr_nr_mas_11_1 F4
PA14_09790 (1)	Transcriptional regulator	PAMr_nr_mas_04_1 E9
PA14_09790 (2)	Transcriptional regulator	PAMr_nr_mas_09_4 E10
PA14_09790 (3)	Transcriptional regulator	PAMr_nr_mas_15_1 A8
PA14_09910	Transcriptional regulator	PAMr_nr_mas_09_4 E1
PA14_09960	Transcriptional regulator	PAMr_nr_mas_08_2 H2
PA14_10010	Transcriptional regulator	PAMr_nr_mas_13_2 D1
PA14_10090	Transcriptional regulator	PAMr_nr_mas_04_1 C3
PA14_10120	Transcriptional regulator	PAMr_nr_mas_01_1 C4
PA14_10190	Transcriptional regulator	PAMr_nr_mas_08_4 F10
PA14_10290 (*acoR*)	Transcriptional regulator	PAMr_nr_mas_05_4 D5
PA14_10320	Transcriptional regulator	PAMr_nr_mas_09_4 H2
PA14_10530 (1)	Transcriptional regulator	PAMr_nr_mas_05_2 C12
PA14_10530 (2)	Transcriptional regulator	PAMr_nr_mas_07_1 F10

PA14_10660	Transcriptional regulator	PAMr_nr_mas_08_2 C8
PA14_10670 (*aph*)	Phosphotransferase	PAMr_nr_mas_08_3 B5
PA14_10700 (1)	Two-component system	PAMr_nr_mas_03_3 G2
PA14_10700 (2)	Two-component system	PAMr_nr_mas_04_2 G6
PA14_10770 (1)	Two-component system	PAMr_nr_mas_08_1 B5
PA14_10770 (2)	Two-component system	PAMr_nr_mas_13_2 F3
PA14_10770 (3)	Two-component system	PAMr_nr_mas_14_4 A12
PA14_10800 (*ampR*)	Transcriptional regulator	ExMr_nr_mas_01_1 B5
PA14_10830	Transcriptional regulator	PAMr_nr_mas_07_4 C5
PA14_10940	Transcriptional regulator	PAMr_nr_mas_08_4 A9
PA14_10980	Transcriptional regulator	PAMr_nr_mas_11_1 A10
PA14_11120	Two-component system	PAMr_nr_mas_08_4 D4
PA14_11180	Transcriptional regulator	PAMr_nr_mas_05_3 D7
PA14_11240 (1)	Transcriptional regulator	PAMr_nr_mas_11_1 B2
PA14_11240 (2)	Transcriptional regulator	PAMr_nr_mas_14_4 B1
PA14_11630	Two-component system	PAMr_nr_mas_09_3 F9
PA14_11680	Two-component system	PAMr_nr_mas_02_2 C1
PA14_11730	Serine/Threonine kinase	PAMr_nr_mas_12_2 H8
PA14_11830	Transcriptional regulator	PAMr_nr_mas_11_2 E4
PA14_12140	Transcriptional regulator	PAMr_nr_mas_05_4 E3
PA14_12440 (1)	Transcriptional regulator	ExMr_nr_mas_01_2 C11
PA14_12440 (2)	Transcriptional regulator	PAMr_nr_mas_13_2 B1
PA14_12570	Transcriptional regulator	PAMr_nr_mas_02_4 A5
PA14_12780	Two-component system	PAMr_nr_mas_12_2 A9
PA14_12810 (1)	Two-component system	PAMr_nr_mas_12_1 F5
PA14_12810 (2)	Two-component system	PAMr_nr_mas_14_2 D11
PA14_12810 (3)	Two-component system	PAMr_nr_mas_14_4 G4
PA14_12820	Two-component system	PAMr_nr_mas_09_4 F2
PA14_13000	Transcriptional regulator	PAMr_nr_mas_12_4 E4
PA14_13060	Transcriptional regulator	PAMr_nr_mas_01_1 D2
PA14_13090 (1)	Two-component system	PAMr_nr_mas_03_3 D12
PA14_13090 (2)	Two-component system	PAMr_nr_mas_15_3 F4
PA14_13150 (1)	Transcriptional regulator	PAMr_nr_mas_12_1 C1
PA14_13150 (2)	Transcriptional regulator	PAMr_nr_mas_15_1 H4
PA14_13450	Transmembrane sensor	PAMr_nr_mas_09_1 E2
PA14_13470	Transcriptional regulator	PAMr_nr_mas_13_4 A1
PA14_13510	Transcriptional regulator	PAMr_nr_mas_02_1 G8
PA14_13660 (*tpbA*)	Two-component system	PAMr_nr_mas_02_1 H7
PA14_13740 (*narX*)	Two-component system	PAMr_nr_mas_10_3 B1
PA14_13780 (*narG*)	Two-component system	PAMr_nr_mas_01_1 C10
PA14_13800 (*narH*) (1)	Two-component system	PAMr_nr_mas_03_3 F1
PA14_13800 (*narH*) (2)	Two-component system	PAMr_nr_mas_10_4 A11

PA14_13810 (*narJ*)	Two-component system	PAMr_nr_mas_01_4 E3
PA14_13830 (*narI*)	Two-component system	PAMr_nr_mas_09_4 C9
PA14_14280	Transcriptional regulator	PAMr_nr_mas_10_4 A6
PA14_14480	Transcriptional regulator	PAMr_nr_mas_01_1 A11
PA14_14710	Transcriptional regulator	PAMr_nr_mas_07_3 E9
PA14_15150	Transcriptional regulator	PAMr_nr_mas_03_1 C1
PA14_15290	Two-component system	PAMr_nr_mas_06_2 F7
PA14_15450 (*merD*) (1)	Transcriptional regulator	PAMr_nr_mas_05_1 F7
PA14_15450 (*merD*) (2)	Transcriptional regulator	PAMr_nr_mas_15_3 D1
PA14_15650	Transcriptional regulator	PAMr_nr_mas_01_4 C8
PA14_15830	Transcriptional regulator	PAMr_nr_mas_05_4 H6
PA14_16280	Transcriptional regulator	PAMr_nr_mas_05_1 B6
PA14_16350	Two-component system	PAMr_nr_mas_06_1 A11
PA14_16440	Two-component system	PAMr_nr_mas_02_1 A4
PA14_16450 (*wspC*)	Two-component system	ExMr_nr_mas_01_2 A2
PA14_16460 (*wspD*)	Two-component system	ExMr_nr_mas_01_1 F2
PA14_16470	Two-component system	PAMr_nr_mas_02_2 C11
PA14_16480 (*wspF*)	Two-component system	PAMr_nr_mas_04_1 C7
PA14_16500 (*wspR*)	Two-component system	PAMr_nr_mas_03_3 H5
PA14_16550	Transcriptional regulator	PAMr_nr_mas_07_4 G2
PA14_16670	Transcriptional regulator	PAMr_nr_mas_12_1 F6
PA14_16790	Transcriptional regulator	PAMr_nr_mas_12_3 G2
PA14_17040 (*glnD*)	Two-component system	PAMr_nr_mas_11_3 G11
PA14_17380	Transcriptional regulator	PAMr_nr_mas_05_3 H8
PA14_17480 (*rpoS*)	Sigma factor	PAMr_nr_mas_05_3 A2
PA14_17670	Two-component system	PAMr_nr_mas_11_2 D1
PA14_17720	Transcriptional regulator	PAMr_nr_mas_13_1 B2
PA14_17900 (*metR*)	Transcriptional regulator	PAMr_nr_mas_12_1 F10
PA14_17990	Predicted aminoacyl-tRNA synthetase	ExMr_nr_mas_01_1 G9
PA14_18080	Transcriptional regulator	PAMr_nr_mas_03_2 D1
PA14_18110 (*mmsR*) (1)	Transcriptional regulator	ExMr_nr_mas_01_1 F12
PA14_18110 (*mmsR*) (2)	Transcriptional regulator	PAMr_nr_mas_02_2 G4
PA14_18200	Transcriptional regulator	PAMr_nr_mas_14_2 F1
PA14_18230 (*fruR*) (1)	Transcriptional regulator	PAMr_nr_mas_01_2 C7
PA14_18230 (*fruR*) (2)	Transcriptional regulator	PAMr_nr_mas_04_4 C4
PA14_18370	Two-component system	PAMr_nr_mas_12_1 F8
PA14_18580 (*algD*)	Two-component system	PAMr_nr_mas_13_3 B12
PA14_19120 (*rhlR*)	Transcriptional regulator	PAMr_nr_mas_08_1 G7
PA14_19190 (1)	Two-component system	PAMr_nr_mas_01_1 H7
PA14_19190 (2)	Two-component system	PAMr_nr_mas_07_4 D9
PA14_19340	Two-component system	PAMr_nr_mas_04_2 D6
PA14_19430	Two-component system	PAMr_nr_mas_11_3 E8

PA14_19670	Transcriptional regulator	ExMr_nr_mas_01_2 F8
PA14_19800 (1)	Transcriptional regulator	PAMr_nr_mas_04_3 D2
PA14_19800 (2)	Transcriptional regulator	PAMr_nr_mas_13_2 G5
PA14_19850	Transcriptional regulator	PAMr_nr_mas_07_2 F2
PA14_20230 (*nosR*)	Transcriptional regulator	PAMr_nr_mas_12_3 G7
PA14_20580 (*amiC*)	Transcriptional regulator	PAMr_nr_mas_07_2 D9
PA14_20670	Two-component system	PAMr_nr_mas_13_1 G7
PA14_20730 (*flgM*) (1)	Sigma factor	PAMr_nr_mas_13_2 F4
PA14_20730 (*flgM*) (2)	Sigma factor	PAMr_nr_mas_13_2 H6
PA14_20750	Two-component system	PAMr_nr_mas_02_3 H4
PA14_20760	Two-component system	PAMr_nr_mas_07_4 A4
PA14_20770	Sigma factor	PAMr_nr_mas_09_1 D11
PA14_20780	Two-component system	PAMr_nr_mas_05_1 C6
PA14_20800 (1)	Two-component system	PAMr_nr_mas_02_4 H3
PA14_20800 (2)	Two-component system	PAMr_nr_mas_03_2 D4
PA14_20820 (1)	Two-component system	PAMr_nr_mas_12_3 G1
PA14_20820 (2)	Two-component system	PAMr_nr_mas_14_4 E10
PA14_20850	Transcriptional regulator	PAMr_nr_mas_08_3 E5
PA14_21080	Transcriptional regulator	PAMr_nr_mas_07_4 B5
PA14_21410 (*phoA*) (1)	Two-component system	PAMr_nr_mas_07_2 C2
PA14_21410 (*phoA*) 2	Two-component system	PAMr_nr_mas_13_2 D3
PA14_21700 (1)	Two-component system	PAMr_nr_mas_07_2 C4
PA14_21700 (2)	Two-component system	PAMr_nr_mas_15_2 B12
PA14_21720	Transcriptional regulator	PAMr_nr_mas_11_3 B6
PA14_21850	Transcriptional regulator	PAMr_nr_mas_13_4 B3
PA14_21970	Transcriptional regulator	PAMr_nr_mas_02_2 F7
PA14_22370 (1)	Two-component system	PAMr_nr_mas_01_3 E4
PA14_22370 (2)	Two-component system	PAMr_nr_mas_13_4 A8
PA14_22470	Transcriptional regulator	PAMr_nr_mas_03_4 G9
PA14_22550 (1)	Transcriptional regulator	PAMr_nr_mas_13_1 G1
PA14_22550 (2)	Transcriptional regulator	PAMr_nr_mas_15_2 F5
PA14_22640 (1)	Transcriptional regulator	PAMr_nr_mas_05_3 H7
PA14_22640 (2)	Transcriptional regulator	PAMr_nr_mas_15_2 E3
PA14_22730	Two-component system	PAMr_nr_mas_03_2 E2
PA14_22860	Transcriptional regulator	PAMr_nr_mas_01_1 B11
PA14_22940 (*gltR*)	Two-component system	PAMr_nr_mas_06_2 D3
PA14_22960	Two-component system	PAMr_nr_mas_04_2 C9
PA14_23060 (*hexR*) (1)	Transcriptional regulator	PAMr_nr_mas_01_3 C9
PA14_23060 (*hexR*) (2)	Transcriptional regulator	PAMr_nr_mas_10_3 F2
PA14_23130	Two-component system	PAMr_nr_mas_04_2 A9
PA14_23190	Transcriptional regulator	PAMr_nr_mas_11_4 G7
PA14_23540 (*act*)	Transcriptional regulator	PAMr_nr_mas_03_3 F6

PA14_23700	Transcriptional regulator	PAMr_nr_mas_05_1 F11
PA14_23720	Translation initiation inhibitor	PAMr_nr_mas_01_2 G10
PA14_23730	Transcriptional regulator	PAMr_nr_mas_13_1 G10
PA14_24140	Transcriptional regulator	PAMr_nr_mas_02_3 F4
PA14_24340	Two-component system	PAMr_nr_mas_12_3 C1
PA14_24350 (1)	Two-component system	PAMr_nr_mas_03_1 C5
PA14_24350 (2)	Two-component system	PAMr_nr_mas_15_2 B5
PA14_24710	Two-component system	PAMr_nr_mas_02_4 F2
PA14_24720	Two-component system	PAMr_nr_mas_12_2 H12
PA14_24920	Transcriptional regulator	PAMr_nr_mas_05_2 F7
PA14_25800	Transcriptional regulator	PAMr_nr_mas_02_4 E8
PA14_26140	Transcriptional regulator	PAMr_nr_mas_03_2 H12
PA14_26150	Transcriptional regulator	PAMr_nr_mas_12_4 G4
PA14_26330 (1)	Transcriptional regulator	PAMr_nr_mas_01_3 F8
PA14_26330 (2)	Transcriptional regulator	PAMr_nr_mas_05_1 H9
PA14_26570	Transcriptional regulator	PAMr_nr_mas_08_1 B3
PA14_26590 (1)	Transcriptional regulator	PAMr_nr_mas_08_3 A7
PA14_26590 (2)	Transcriptional regulator	PAMr_nr_mas_15_3 F8
PA14_26600	Sigma factor	PAMr_nr_mas_01_1 C12
PA14_26760	Transcriptional regulator	PAMr_nr_mas_03_1 G12
PA14_26810 (1)	Two-component system	PAMr_nr_mas_09_3 F11
PA14_26810 (2)	Two-component system	PAMr_nr_mas_14_1 B1
PA14_26860	Transcriptional regulator	PAMr_nr_mas_10_3 F6
PA14_26880	Transcriptional regulator	PAMr_nr_mas_03_1 D4
PA14_27230 (*ohrR*)	Transcriptional regulator	PAMr_nr_mas_14_1 H5
PA14_27250 (1)	Transcriptional regulator	PAMr_nr_mas_01_4 C12
PA14_27250 (2)	Transcriptional regulator	PAMr_nr_mas_02_1 C1
PA14_27280	Transcriptional regulator	PAMr_nr_mas_03_2 C6
PA14_27400	Transcriptional regulator	PAMr_nr_mas_04_4 B4
PA14_27440 (1)	Transcriptional regulator	PAMr_nr_mas_07_4 D7
PA14_27440 (2)	Transcriptional regulator	PAMr_nr_mas_15_3 C10
PA14_27550	Two-component system	PAMr_nr_mas_08_3 E4
PA14_27570	Two-component system	PAMr_nr_mas_02_1 C11
PA14_27700	Transcriptional regulator	PAMr_nr_mas_05_3 H5
PA14_27800 (1)	Two-component system	PAMr_nr_mas_06_1 G6
PA14_27800 (2)	Two-component system	PAMr_nr_mas_09_3 C2
PA14_27810 (1)	Two-component system	PAMr_nr_mas_07_3 C3
PA14_27810 (2)	Two-component system	PAMr_nr_mas_10_1 D12
PA14_27900	Transcriptional regulator	PAMr_nr_mas_03_2 A4
PA14_27940	Two-component system	PAMr_nr_mas_09_4 A3
PA14_27950	Sigma factor	PAMr_nr_mas_11_2 E2
PA14_28080	Transcriptional regulator	PAMr_nr_mas_11_1 D1

PA14_28130 (1)	Transcriptional regulator	PAMr_nr_mas_01_4 C4
PA14_28130 (2)	Transcriptional regulator	PAMr_nr_mas_14_4 A11
PA14_28320	Transcriptional regulator	PAMr_nr_mas_02_2 G12
PA14_28420	Transcriptional regulator	PAMr_nr_mas_09_4 H6
PA14_29260	Transcriptional regulator	PAMr_nr_mas_04_3 G9
PA14_29300	Transcriptional regulator	PAMr_nr_mas_04_2 B9
PA14_29360 (*pfeS*)	Two-component system	PAMr_nr_mas_12_3 E12
PA14_29590	Transcriptional regulator	PAMr_nr_mas_10_2 B10
PA14_29620 (1)	Transcriptional regulator	PAMr_nr_mas_01_2 E8
PA14_29620 (2)	Transcriptional regulator	PAMr_nr_mas_10_4 G2
PA14_29660	Predicted NnrS protein	PAMr_nr_mas_11_1 A1
PA14_29740	Two-component system	PAMr_nr_mas_09_4 C6
PA14_30580	Transcriptional regulator	PAMr_nr_mas_12_4 C2
PA14_30620	Transcriptional regulator	PAMr_nr_mas_02_1 C5
PA14_30650 (*gacA*) (1)	Two-component system	PAMr_nr_mas_06_3 G12
PA14_30650 (*gacA*) (2)	Two-component system	PAMr_nr_mas_15_3 F7
PA14_30700	Two-component system	PAMr_nr_mas_14_1 A6
PA14_30830	Two-component system	PAMr_nr_mas_13_1 G6
PA14_30840	Two-component system	PAMr_nr_mas_05_2 G6
PA14_30970 (1)	Transcriptional regulator	PAMr_nr_mas_10_2 C11
PA14_30970 (2)	Transcriptional regulator	PAMr_nr_mas_14_4 F1
PA14_31110	Transcriptional regulator	PAMr_nr_mas_09_2 D3
PA14_31380	Sigma factor	PAMr_nr_mas_09_3 B5
PA14_31480	Transcriptional regulator	PAMr_nr_mas_08_4 F7
PA14_31560 (1)	Transcriptional regulator	PAMr_nr_mas_05_4 A6
PA14_31560 (2)	Transcriptional regulator	PAMr_nr_mas_10_3 F12
PA14_31620	Two-component system	PAMr_nr_mas_01_4 H7
PA14_31630	Transcriptional regulator	PAMr_nr_mas_09_4 C4
PA14_31780	Transcriptional regulator	PAMr_nr_mas_08_4 D8
PA14_31870	Two-component system	PAMr_nr_mas_06_1 A4
PA14_31890 (1)	Two-component system	PAMr_nr_mas_06_4 H11
PA14_31890 (2)	Two-component system	PAMr_nr_mas_08_2 E2
PA14_31900	Two-component system	PAMr_nr_mas_04_2 F10
PA14_31950	Two-component system	PAMr_nr_mas_04_3 E4
PA14_31960	Two-component system	PAMr_nr_mas_05_4 E6
PA14_32060 (*xylS*) (1)	Transcriptional regulator	PAMr_nr_mas_09_1 C6
PA14_32060 (*xylS*) (2)	Transcriptional regulator	PAMr_nr_mas_14_1 F6
PA14_32300	Two-component system	PAMr_nr_mas_06_2 C9
PA14_32360	Transcriptional regulator	PAMr_nr_mas_12_2 B10
PA14_32410 (*mexT*)	Transcriptional regulator	PAMr_nr_mas_12_1 E6
PA14_32450	Transcriptional regulator	PAMr_nr_mas_11_2 F8
PA14_32460	Transcriptional regulator	PAMr_nr_mas_02_4 G7

PA14_32500 (1)	Transcriptional regulator	PAMr_nr_mas_01_4 A2
PA14_32500 (2)	Transcriptional regulator	PAMr_nr_mas_11_3 D7
PA14_32580	Two-component system	PAMr_nr_mas_06_4 C10
PA14_32700	Transcriptional regulator	PAMr_nr_mas_05_2 F11
PA14_32940 (1)	Transcriptional regulator	PAMr_nr_mas_06_1 E9
PA14_32940 (2)	Transcriptional regulator	PAMr_nr_mas_08_1 H7
PA14_32970	Transcriptional regulator	ExMr_nr_mas_01_2 E2
PA14_33170 (1)	Transcriptional regulator	PAMr_nr_mas_02_2 D4
PA14_33170 (2)	Transcriptional regulator	PAMr_nr_mas_04_2 B4
PA14_33260 (*pvdS*)	Sigma factor	PAMr_nr_mas_06_2 G5
PA14_33780	Transmembrane sensor	PAMr_nr_mas_06_3 C3
PA14_34150	Transcriptional regulator	PAMr_nr_mas_11_1 C6
PA14_34210	Transcriptional regulator	PAMr_nr_mas_04_1 A1
PA14_34440 (*mtlR*)	Transcriptional regulator	PAMr_nr_mas_03_3 B12
PA14_34660 (*gntR*)	Transcriptional regulator	PAMr_nr_mas_04_4 G10
PA14_34690	Transcriptional regulator	PAMr_nr_mas_07_3 A9
PA14_34730	Transcriptional regulator	PAMr_nr_mas_06_1 B8
PA14_34820	Transcriptional regulator	ExMr_nr_mas_01_2 G11
PA14_34830	Transcriptional regulator	PAMr_nr_mas_13_1 H2
PA14_35070 (1)	Transcriptional regulator	PAMr_nr_mas_01_3 B6
PA14_35070 (2)	Transcriptional regulator	PAMr_nr_mas_14_4 F7
PA14_35130 (*arsR*)	Transcriptional regulator	PAMr_nr_mas_09_2 E6
PA14_35140	Transcriptional regulator	PAMr_nr_mas_14_1 H9
PA14_35380 (*ptxR*)	Transcriptional regulator	PAMr_nr_mas_05_2 F9
PA14_35540 (*bkdR*)	Transcriptional regulator	PAMr_nr_mas_11_4 E11
PA14_35990	Phosphotransferase	PAMr_nr_mas_13_1 E9
PA14_36000 (*prpR*)	Transcriptional regulator	PAMr_nr_mas_01_3 A8
PA14_36180	Transcriptional regulator	PAMr_nr_mas_03_4 E6
PA14_36260	Two-component system	PAMr_nr_mas_01_1 E12
PA14_36300 (*nemR*)	Transcriptional regulator	PAMr_nr_mas_06_4 E6
PA14_36420	Two-component system	PAMr_nr_mas_09_1 B8
PA14_36810 (*katE*) (1)	Catalase	PAMr_nr_mas_09_2 A10
PA14_36810 (*katE*) (2)	Catalase	PAMr_nr_mas_13_1 F6
PA14_36990	Two-component system	PAMr_nr_mas_02_3 H6
PA14_37080	Transcriptional regulator	PAMr_nr_mas_06_1 G7
PA14_37120	Transcriptional regulator	PAMr_nr_mas_13_3 A12
PA14_37140	Transcriptional regulator	PAMr_nr_mas_06_4 H12
PA14_37660 (1)	Transcriptional regulator	PAMr_nr_mas_02_3 H5
PA14_37660 (2)	Transcriptional regulator	PAMr_nr_mas_15_3 G9
PA14_37690 (1)	Two-component system	PAMr_nr_mas_02_4 E10
PA14_37690 (2)	Two-component system	PAMr_nr_mas_14_1 B2
PA14_37940 (*cynR*)	Transcriptional regulator	PAMr_nr_mas_13_2 E10

PA14_37980 (1)	Fe(2+)-dicitrate sensor	PAMr_nr_mas_08_2 A7
PA14_37980 (2)	Fe(2+)-dicitrate sensor	PAMr_nr_mas_14_4 G12
PA14_37990	Sigma factor	PAMr_nr_mas_07_1 H8
PA14_38040 (*cmrA*)	Transcriptional regulator	PAMr_nr_mas_02_3 H1
PA14_38140 (1)	Two-component system	PAMr_nr_mas_01_2 D10
PA14_38140 (2)	Two-component system	PAMr_nr_mas_03_3 C1
PA14_38250 (1)	Transcriptional regulator	PAMr_nr_mas_05_4 F6
PA14_38250 (2)	Transcriptional regulator	PAMr_nr_mas_09_3 F3
PA14_38300	Transcriptional regulator	PAMr_nr_mas_04_2 C4
PA14_38380	Transcriptional regulator	PAMr_nr_mas_09_3 H8
PA14_38430	Transcriptional regulator	PAMr_nr_mas_03_1 F3
PA14_38500 (1)	Transcriptional regulator	PAMr_nr_mas_12_3 B7
PA14_38500 (2)	Transcriptional regulator	PAMr_nr_mas_15_3 E10
PA14_38570	Transcriptional regulator	PAMr_nr_mas_11_2 H11
PA14_38610 (1)	Two-component system	PAMr_nr_mas_08_2 H3
PA14_38610 (2)	Two-component system	PAMr_nr_mas_13_1 C10
PA14_38680	Transcriptional regulator	PAMr_nr_mas_02_2 H3
PA14_38900	Two-component system	PAMr_nr_mas_08_2 B2
PA14_39160	Transcriptional regulator	PAMr_nr_mas_07_3 G4
PA14_39300 (*rbsR*)	Transcriptional regulator	PAMr_nr_mas_03_4 F12
PA14_39360 (1)	Transcriptional regulator	PAMr_nr_mas_04_3 C5
PA14_39360 (2)	Transcriptional regulator	PAMr_nr_mas_05_2 B11
PA14_39460	Catalase	PAMr_nr_mas_10_3 D4
PA14_39770	Two-component system	PAMr_nr_mas_02_3 B7
PA14_39800	Sigma factor	ExMr_nr_mas_01_1 H6
PA14_39980 (*qscR*)	Transcriptional regulator	PAMr_nr_mas_10_3 E8
PA14_40380	Transcriptional regulator	PAMr_nr_mas_07_3 D11
PA14_40440	Transcriptional regulator	PAMr_nr_mas_09_2 B1
PA14_40550	Transcriptional regulator	PAMr_nr_mas_06_1 H4
PA14_40570 (1)	Two-component system	PAMr_nr_mas_09_4 E2
PA14_40570 (2)	Two-component system	PAMr_nr_mas_13_4 E11
PA14_40600 (1)	Transcriptional regulator	PAMr_nr_mas_01_3 E1
PA14_40600 (2)	Transcriptional regulator	PAMr_nr_mas_03_3 H8
PA14_40790	Transcriptional regulator	PAMr_nr_mas_08_2 E11
PA14_40880	Phosphotransferase	PAMr_nr_mas_10_3 A12
PA14_40910	Transcriptional regulator	PAMr_nr_mas_10_2 D7
PA14_41220 (*lon*)	Lon serine protease	PAMr_nr_mas_04_1 A9
PA14_41260 (1)	Two-component system	PAMr_nr_mas_13_4 G8
PA14_41260 (2)	Two-component system	PAMr_nr_mas_15_2 D5
PA14_41270	Two-component system	PAMr_nr_mas_03_3 E12
PA14_41480 (*nasS*) (1)	Two-component system	PAMr_nr_mas_01_1 C3
PA14_41480 (*nasS*) (2)	Two-component system	PAMr_nr_mas_01_3 A5

PA14_41490 (*nasT*)	Two-component system	PAMr_nr_mas_10_4 A7
PA14_41520	Serine/Threonine kinase	PAMr_nr_mas_07_1 A5
PA14_41800	Transcriptional regulator	PAMr_nr_mas_09_2 B9
PA14_41810	Transcriptional regulator	PAMr_nr_mas_04_4 C7
PA14_41830 (*thrH*) (1)	Serine/Threonine kinase	ExMr_nr_mas_01_2 C6
PA14_41830 (*thrH*) (2)	Serine/Threonine kinase	PAMr_nr_mas_12_1 B12
PA14_41870 (*cysB*)	Transcriptional regulator	PAMr_nr_mas_10_3 B3
PA14_42060	Transcriptional regulator	PAMr_nr_mas_10_4 H9
PA14_42090	Two-component system	PAMr_nr_mas_08_1 H5
PA14_42220 (1)	Membrane sensor	PAMr_nr_mas_10_2 C5
PA14_42220 (2)	Membrane sensor	PAMr_nr_mas_15_1 B11
PA14_42390 (*exsA*)	Transcriptional regulator	PAMr_nr_mas_01_1 H9
PA14_42460 (*pcrH*)	T3SS regulator	PAMr_nr_mas_12_3 F4
PA14_42880 (*stk1*)	Serine/Threonine kinase	PAMr_nr_mas_06_1 F1
PA14_42970	Transcriptional regulator	PAMr_nr_mas_04_3 H5
PA14_43200	Sigma factor	PAMr_nr_mas_06_4 A5
PA14_43240	Predicted Flavin reductase	PAMr_nr_mas_04_4 C2
PA14_43350 (*kdpD*) (1)	Two-component system	PAMr_nr_mas_05_2 A3
PA14_43350 (*kdpD*) (2)	Two-component system	PAMr_nr_mas_12_4 H3
PA14_43380 (*kdpB*) (1)	Two-component system	ExMr_nr_mas_01_1 E7
PA14_43380 (*kdpB*) (2)	Two-component system	PAMr_nr_mas_06_2 F6
PA14_43400 (*kdpA*) (1)	Two-component system	PAMr_nr_mas_06_2 F2
PA14_43400 (*kdpA*) (2)	Two-component system	PAMr_nr_mas_09_1 C5
PA14_43430	Transcriptional regulator	PAMr_nr_mas_09_3 D10
PA14_43480	Transcriptional regulator	PAMr_nr_mas_07_2 A7
PA14_43670 (1)	Two-component system	ExMr_nr_mas_01_2 A5
PA14_43670 (2)	Two-component system	PAMr_nr_mas_07_3 F3
PA14_43720	Transcriptional regulator	PAMr_nr_mas_02_4 F5
PA14_43770	Transcriptional regulator	PAMr_nr_mas_08_4 F4
PA14_44180	Transcriptional regulator	PAMr_nr_mas_12_1 F2
PA14_44240	Two-component system	PAMr_nr_mas_08_4 F6
PA14_44300 (*aer*)	Two-component system	PAMr_nr_mas_13_2 E11
PA14_44490 (*anr*)	Transcriptional regulator	PAMr_nr_mas_02_4 D9
PA14_44690 (1)	Transcriptional regulator	PAMr_nr_mas_12_2 A5
PA14_44690 (2)	Transcriptional regulator	PAMr_nr_mas_15_2 A9
PA14_44980	Transcriptional regulator	PAMr_nr_mas_08_3 D8
PA14_45250	Transcriptional regulator	PAMr_nr_mas_10_4 C2
PA14_45460	Transcriptional regulator	PAMr_nr_mas_06_1 A7
PA14_45500	Two-component system	PAMr_nr_mas_11_4 D3
PA14_45510	Two-component system	PAMr_nr_mas_08_2 A9
PA14_45560 (*motC*)	Two-component system	PAMr_nr_mas_04_3 B8
PA14_45580	Two-component system	PAMr_nr_mas_06_4 F6

PA14_45590 (1)	Two-component system	ExMr_nr_mas_01_2 B2
PA14_45590 (2)	Two-component system	PAMr_nr_mas_06_2 D4
PA14_45590 (3)	Two-component system	PAMr_nr_mas_13_3 E4
PA14_45630 (*fliA*) (1)	Sigma factor	PAMr_nr_mas_03_3 E6
PA14_45630 (*fliA*) (2)	Sigma factor	PAMr_nr_mas_14_4 D8
PA14_45640 (*fleN*)	Flagellar synthesis regulator	PAMr_nr_mas_01_4 G10
PA14_45870 (1)	Two-component system	PAMr_nr_mas_05_1 A12
PA14_45870 (2)	Two-component system	PAMr_nr_mas_14_4 B4
PA14_45880	Two-component system	PAMr_nr_mas_07_2 B11
PA14_45950 (*rsaL*)	Transcriptional regulator	PAMr_nr_mas_12_1 C8
PA14_46060 (*gbuR*)	Transcriptional regulator	PAMr_nr_mas_03_2 F4
PA14_46290	Transcriptional regulator	PAMr_nr_mas_01_2 F5
PA14_46330	Transcriptional regulator	PAMr_nr_mas_04_4 G3
PA14_46370	Two-component system	PAMr_nr_mas_01_1 H6
PA14_46400	Transcriptional regulator	PAMr_nr_mas_09_2 H11
PA14_46480	Transcriptional regulator	PAMr_nr_mas_02_3 A4
PA14_46570	Transcriptional regulator	PAMr_nr_mas_10_3 C9
PA14_46810	Sigma factor	PAMr_nr_mas_07_1 E11
PA14_46850	Transcriptional regulator	PAMr_nr_mas_04_3 E6
PA14_46980	Two-component system	PAMr_nr_mas_03_2 H8
PA14_47240 (1)	Transcriptional regulator	PAMr_nr_mas_12_1 F4
PA14_47240 (2)	Transcriptional regulator	PAMr_nr_mas_15_3 H3
PA14_47270 (1)	Transcriptional regulator	PAMr_nr_mas_02_2 H4
PA14_47270 (2)	Transcriptional regulator	PAMr_nr_mas_14_4 B12
PA14_47390	Two-component system	PAMr_nr_mas_09_2 E7
PA14_47520	Transcriptional regulator	PAMr_nr_mas_02_2 G8
PA14_47580	Transcriptional regulator	PAMr_nr_mas_12_2 H7
PA14_47820	Transcriptional regulator	PAMr_nr_mas_14_1 E10
PA14_47880	Transcriptional regulator	PAMr_nr_mas_01_3 H7
PA14_47910	Transcriptional regulator	PAMr_nr_mas_03_4 E7
PA14_48160	Two-component system	PAMr_nr_mas_09_1 H11
PA14_48190	Transcriptional regulator	PAMr_nr_mas_10_2 G4
PA14_48390 (1)	Transcriptional regulator	ExMr_nr_mas_01_2 D6
PA14_48390 (2)	Transcriptional regulator	PAMr_nr_mas_05_2 D10
PA14_48390 (3)	Transcriptional regulator	PAMr_nr_mas_07_4 H2
PA14_48410	Serine/Threonine kinase	PAMr_nr_mas_11_4 D10
PA14_48420	Transcriptional regulator	PAMr_nr_mas_09_1 F12
PA14_48770 (1)	Transcriptional regulator	PAMr_nr_mas_01_1 D10
PA14_48770 (2)	Transcriptional regulator	PAMr_nr_mas_15_3 A2
PA14_48810	Transcriptional regulator	PAMr_nr_mas_14_2 B5
PA14_48830 (1)	Transcriptional regulator	PAMr_nr_mas_03_4 C9
PA14_48830 (2)	Transcriptional regulator	PAMr_nr_mas_04_1 E11

PA14_49130 (*dctA*)	Two-component system	PAMr_nr_mas_10_1 F7
PA14_49170 (*phoQ*)	Two-component system	PAMr_nr_mas_05_4 G3
PA14_49180 (*phoP*)	Two-component system	PAMr_nr_mas_09_3 A3
PA14_49420	Two-component system	PAMr_nr_mas_03_4 E10
PA14_49440 (1)	Two-component system	PAMr_nr_mas_02_3 B11
PA14_49440 (2)	Two-component system	PAMr_nr_mas_08_4 B5
PA14_49590	Transcriptional regulator	PAMr_nr_mas_05_2 C9
PA14_49640	Transcriptional regulator	PAMr_nr_mas_02_2 C7
PA14_49680 (1)	Transcriptional regulator	PAMr_nr_mas_02_2 F3
PA14_49680 (2)	Transcriptional regulator	PAMr_nr_mas_06_4 B10
PA14_49790	Transcriptional regulator	PAMr_nr_mas_11_4 G12
PA14_49890 (*tpbB*)	Two-component system	PAMr_nr_mas_08_1 D6
PA14_50040 (1)	Transcriptional regulator	PAMr_nr_mas_04_1 G9
PA14_50040 (2)	Transcriptional regulator	PAMr_nr_mas_15_1 D3
PA14_50180 (*fleR*) (1)	Two-component system	PAMr_nr_mas_04_1 F11
PA14_50180 (*fleR*) (2)	Two-component system	PAMr_nr_mas_05_4 G11
PA14_50200 (*fleS*) (1)	Two-component system	PAMr_nr_mas_10_3 C5
PA14_50200 (*fleS*) (2)	Two-component system	PAMr_nr_mas_12_4 F12
PA14_50220 (*fleQ*) (1)	Transcriptional regulator	PAMr_nr_mas_01_3 H6
PA14_50220 (*fleQ*) (2)	Transcriptional regulator	PAMr_nr_mas_10_1 A1
PA14_50290 (*fliC*)	Two-component system	PAMr_nr_mas_07_3 A8
PA14_50600	Transcriptional regulator	PAMr_nr_mas_13_2 H11
PA14_51280	Transcriptional regulator	PAMr_nr_mas_04_3 F3
PA14_51340 (*mvfR*)	Transcriptional regulator	PAMr_nr_mas_12_1 G5
PA14_51360 (*phnA*) (1)	Two-component system	PAMr_nr_mas_04_1 H11
PA14_51360 (*phnA*) (2)	Two-component system	PAMr_nr_mas_11_3 C1
PA14_51990 (*wrbA*)	Trp repressor binding protein	PAMr_nr_mas_07_4 D2
PA14_52070 (*pqrR*)	Transcriptional regulator	PAMr_nr_mas_02_3 G11
PA14_52240	Two-component system	PAMr_nr_mas_05_4 B10
PA14_52250	Two-component system	ExMr_nr_mas_01_2 E7
PA14_52260 (1)	Two-component system	PAMr_nr_mas_12_1 G4
PA14_52260 (2)	Two-component system	PAMr_nr_mas_15_2 H9
PA14_52570 (*rsmA*)	Transcriptional regulator	PAMr_nr_mas_11_2 A2
PA14_52740 (*argR*)	Transcriptional regulator	PAMr_nr_mas_04_2 G8
PA14_52810	Two-component system	PAMr_nr_mas_11_1 E9
PA14_52820	Two-component system	PAMr_nr_mas_14_2 A4
PA14_52930	Transcriptional regulator	PAMr_nr_mas_05_3 F9
PA14_52980 (*phhR*)	Transcriptional regulator	PAMr_nr_mas_01_3 D6
PA14_53090	Transcriptional regulator	PAMr_nr_mas_11_4 A12
PA14_53410 (1)	Transcriptional regulator	PAMr_nr_mas_13_1 E10
PA14_53410 (2)	Transcriptional regulator	PAMr_nr_mas_14_4 B9
PA14_53520 (*oruR*)	Transcriptional regulator	PAMr_nr_mas_04_1 E10

PA14_53550	Transcriptional regulator	PAMr_nr_mas_09_1 A3
PA14_53730	Transcriptional regulator	PAMr_nr_mas_06_4 C7
PA14_53920	Transcriptional regulator	PAMr_nr_mas_14_1 B6
PA14_54010	Transcriptional regulator	PAMr_nr_mas_03_1 D5
PA14_54130	Transcriptional regulator	PAMr_nr_mas_08_3 H9
PA14_54410 (*mucB*) (1)	Transcriptional regulator	ExMr_nr_mas_01_1 F9
PA14_54410 (*mucB*) (2)	Transcriptional regulator	PAMr_nr_mas_02_1 G6
PA14_54430 (*algU*)	Sigma factor	PAMr_nr_mas_09_3 C3
PA14_54500	Two-component system	PAMr_nr_mas_08_1 F6
PA14_54510	Two-component system	PAMr_nr_mas_02_4 A4
PA14_54540	Two-component system	PAMr_nr_mas_02_3 B1
PA14_54570	Two-component system	PAMr_nr_mas_04_4 C8
PA14_54610	Transcriptional regulator	PAMr_nr_mas_12_2 C11
PA14_55150	Transcriptional regulator	PAMr_nr_mas_10_1 F10
PA14_55250	Transcriptional regulator	PAMr_nr_mas_01_1 D8
PA14_55730	Transcriptional regulator	PAMr_nr_mas_08_2 H7
PA14_55780	Two-component system	PAMr_nr_mas_11_4 B9
PA14_55810	Two-component system	PAMr_nr_mas_08_3 C3
PA14_56070 (*mvaT*)	Transcriptional regulator	PAMr_nr_mas_06_3 B7
PA14_56430	Transcriptional regulator	PAMr_nr_mas_13_3 E7
PA14_56620 (*pyeR*) (1)	Transcriptional regulator	ExMr_nr_mas_01_1 C5
PA14_56620 (*pyeR*) (2)	Transcriptional regulator	PAMr_nr_mas_11_4 A10
PA14_56790	Two-component system	PAMr_nr_mas_12_3 H6
PA14_56950	Two-component system	PAMr_nr_mas_02_2 H8
PA14_57140	Two-component system	PAMr_nr_mas_04_4 C12
PA14_57170	Two-component system	PAMr_nr_mas_13_3 A6
PA14_57630	Transcriptional regulator	PAMr_nr_mas_11_3 C12
PA14_57940 (*rpoN*) (1)	Sigma factor	PAMr_nr_mas_11_1 H10
PA14_57940 (*rpoN*) (2)	Sigma factor	PAMr_nr_mas_14_3 B11
PA14_58000 (*sodM*)	Superoxide Dismutase	PAMr_nr_mas_07_3 B5
PA14_58300	Two-component system	PAMr_nr_mas_09_3 E8
PA14_58320 (1)	Two-component system	PAMr_nr_mas_06_1 C4
PA14_58320 (2)	Two-component system	PAMr_nr_mas_09_4 F8
PA14_58380	Transcriptional regulator	PAMr_nr_mas_12_2 A8
PA14_58670 (*ampD*)	β-lactamase repressor	PAMr_nr_mas_10_4 A4
PA14_59240 (*piL2*)	Two-component system	ExMr_nr_mas_01_1 F10
PA14_59770 (*rcsB*)	Two-component system	PAMr_nr_mas_10_2 E11
PA14_59780 (*rcsC*) (1)	Two-component system	PAMr_nr_mas_05_3 D6
PA14_59780 (*rcsC*) (2)	Two-component system	PAMr_nr_mas_15_1 G10
PA14_59790 (*pvrR*) (1)	Two-component system	PAMr_nr_mas_08_1 E6
PA14_59790 (*pvrR*) (2)	Two-component system	PAMr_nr_mas_15_2 A11
PA14_59800 (*pvrS*) (1)	Two-component system	PAMr_nr_mas_01_4 A5

PA14_59800 (*pvrS*) (2)	Two-component system	PAMr_nr_mas_05_1 G11
PA14_60250 (*pilS*)	Two-component system	PAMr_nr_mas_06_3 G7
PA14_60260 (*pilR*)	Two-component system	PAMr_nr_mas_07_4 F6
PA14_60600 (*rtcR*) (1)	Transcriptional regulator	PAMr_nr_mas_05_4 E7
PA14_60600 (*rtcR*) (2)	Transcriptional regulator	PAMr_nr_mas_13_4 D2
PA14_60860 (*nfxB*)	Transcriptional regulator	PAMr_nr_mas_13_4 A11
PA14_61020 (1)	Two-component system	PAMr_nr_mas_05_4 B12
PA14_61020 (2)	Two-component system	PAMr_nr_mas_14_4 C6
PA14_61040 (*katB*) (1)	Catalase	PAMr_nr_mas_02_3 B12
PA14_61040 (*katB*) (2)	Catalase	PAMr_nr_mas_04_4 G9
PA14_61620	Transcriptional regulator	PAMr_nr_mas_05_4 C5
PA14_62260 (1)	Two-component system	PAMr_nr_mas_07_4 A1
PA14_62260 (2)	Two-component system	PAMr_nr_mas_15_1 F7
PA14_62490 (*dksA*)	Transcriptional regulator	PAMr_nr_mas_10_1 B12
PA14_62530 (*cbrA*) (1)	Two-component system	PAMr_nr_mas_06_2 A7
PA14_62530 (*cbrA*) (2)	Two-component system	PAMr_nr_mas_15_1 F6
PA14_62540 (*cbrB*)	Two-component system	PAMr_nr_mas_14_3 D8
PA14_63070	Transcriptional regulator	PAMr_nr_mas_01_2 G9
PA14_63150 (*pmrA*)	Two-component system	PAMr_nr_mas_07_4 F7
PA14_63160 (*pmrB*) (1)	Two-component system	ExMr_nr_mas_01_2 B4
PA14_63160 (*pmrB*) (2)	Two-component system	PAMr_nr_mas_07_1 B8
PA14_63170	Transcriptional regulator	PAMr_nr_mas_02_3 F5
PA14_63210	Two-component system	PAMr_nr_mas_13_3 H4
PA14_63250 (1)	Two-component system	PAMr_nr_mas_06_3 F11
PA14_63250 (2)	Two-component system	PAMr_nr_mas_15_2 C8
PA14_63280	Transcriptional regulator	PAMr_nr_mas_13_3 A2
PA14_63520	Transcriptional regulator	PAMr_nr_mas_02_3 B4
PA14_63880 (1)	Transcriptional regulator	PAMr_nr_mas_11_2 G7
PA14_63880 (2)	Transcriptional regulator	PAMr_nr_mas_11_3 C7
PA14_64050 (1)	Two-component system	PAMr_nr_mas_11_2 C11
PA14_64050 (2)	Two-component system	PAMr_nr_mas_13_2 G7
PA14_64230 (*retS*)	Two-component system	PAMr_nr_mas_01_4 C2
PA14_64500	Transcriptional regulator	PAMr_nr_mas_13_2 H5
PA14_64640	Transcriptional regulator	PAMr_nr_mas_13_3 C2
PA14_64690	Transmembrane sensor	PAMr_nr_mas_07_4 H3
PA14_64700	Sigma factor	PAMr_nr_mas_01_3 E12
PA14_64780	Transcriptional regulator	PAMr_nr_mas_03_1 G10
PA14_64910	Transcriptional regulator	PAMr_nr_mas_06_4 G3
PA14_65450 (*motA*) (1)	Two-component system	PAMr_nr_mas_03_2 E7
PA14_65450 (*motA*) (2)	Two-component system	PAMr_nr_mas_12_3 D12
PA14_65860	Two-component system	PAMr_nr_mas_04_2 F4

PA14_65880 (1)	Two-component system	PAMr_nr_mas_13_4 D10
PA14_65880 (2)	Two-component system	PAMr_nr_mas_13_4 G7
PA14_65880 (3)	Two-component system	PAMr_nr_mas_15_2 G11
PA14_65900	Transcriptional regulator	PAMr_nr_mas_14_1 H4
PA14_65950	Transcriptional regulator	PAMr_nr_mas_03_2 F7
PA14_65970	Transcriptional regulator	ExMr_nr_mas_01_2 E10
PA14_66320	Two-component system	PAMr_nr_mas_11_4 B4
PA14_66490 (1)	Transcriptional regulator	PAMr_nr_mas_07_1 E10
PA14_66490 (2)	Transcriptional regulator	PAMr_nr_mas_10_2 A12
PA14_66530	Transcriptional regulator	PAMr_nr_mas_05_1 G2
PA14_66850	Transcriptional regulator	PAMr_nr_mas_13_4 F4
PA14_67140	Predicted endoribonuclease	PAMr_nr_mas_12_3 F6
PA14_67170 (1)	Transcriptional regulator	PAMr_nr_mas_12_1 E1
PA14_67170 (2)	Transcriptional regulator	PAMr_nr_mas_13_1 G8
PA14_67170 (3)	Transcriptional regulator	PAMr_nr_mas_15_2 B4
PA14_67550	Transcriptional regulator	PAMr_nr_mas_11_4 G3
PA14_67670 (*ntrB*)	Two-component system	PAMr_nr_mas_12_4 D4
PA14_67680 (*ntrC*) (1)	Two-component system	PAMr_nr_mas_02_4 H1
PA14_67680 (*ntrC*) (2)	Two-component system	PAMr_nr_mas_04_3 E10
PA14_68110 (1)	Transcriptional regulator	PAMr_nr_mas_10_3 F11
PA14_68110 (2)	Transcriptional regulator	PAMr_nr_mas_15_3 A7
PA14_68230 (1)	Two-component system	PAMr_nr_mas_06_1 H2
PA14_68230 (2)	Two-component system	PAMr_nr_mas_15_1 G3
PA14_68250 (1)	Two-component system	PAMr_nr_mas_10_1 G10
PA14_68250 (2)	Two-component system	PAMr_nr_mas_14_1 B7
PA14_68260	Two-component system	PAMr_nr_mas_03_3 G5
PA14_68280 (1)	Two-component system	PAMr_nr_mas_07_1 A7
PA14_68280 (2)	Two-component system	PAMr_nr_mas_14_2 G5
PA14_68290	Two-component system	PAMr_nr_mas_07_3 A12
PA14_68420	Transcriptional regulator	PAMr_nr_mas_12_3 H11
PA14_68550	Transcriptional regulator	PAMr_nr_mas_02_1 F8
PA14_68680 (*envZ*) (1)	Two-component system	ExMr_nr_mas_01_1 B8
PA14_68680 (*envZ*) (2)	Two-component system	PAMr_nr_mas_07_2 G12
PA14_68680 (*envZ*) (3)	Two-component system	PAMr_nr_mas_13_3 C5
PA14_68800 (1)	Putative phosphate transport regulator	PAMr_nr_mas_04_2 B10
PA14_68800 (2)	Putative phosphate transport regulator	PAMr_nr_mas_05_1 E2
PA14_68920 (1)	Transcriptional regulator	PAMr_nr_mas_09_4 F11
PA14_68920 (2)	Transcriptional regulator	PAMr_nr_mas_10_4 B4
PA14_68920 (3)	Transcriptional regulator	PAMr_nr_mas_15_3 A1
PA14_69370 (*algP*) (1)	Transcriptional regulator	PAMr_nr_mas_01_1 E4
PA14_69370 (*algP*) (2)	Transcriptional regulator	PAMr_nr_mas_06_4 B9
PA14_69390 (*algQ*)	Sigma factor	PAMr_nr_mas_09_4 B11

PA14_69470 (*algR*)	Two-component system	PAMr_nr_mas_12_1 D11
PA14_69480 (*algZ*)	Two-component system	PAMr_nr_mas_05_2 B2
PA14_69630 (*rnk*)	Transcriptional regulator	PAMr_nr_mas_04_1 H8
PA14_69750	Transcriptional regulator	PAMr_nr_mas_11_1 B6
PA14_69880	Transcriptional regulator	PAMr_nr_mas_06_2 B12
PA14_69900 (1)	Two-component system	PAMr_nr_mas_01_1 G1
PA14_69900 (2)	Two-component system	PAMr_nr_mas_15_1 B6
PA14_69980	Transcriptional regulator	PAMr_nr_mas_01_4 C10
PA14_70080 (*lrp*)	Transcriptional regulator	PAMr_nr_mas_10_1 C8
PA14_70290	Transcriptional regulator	PAMr_nr_mas_04_4 H10
PA14_70530 (1)	Transcriptional regulator	PAMr_nr_mas_04_1 A8
PA14_70530 (2)	Transcriptional regulator	PAMr_nr_mas_10_3 F5
PA14_70570 (*recG*)	Transcriptional regulator	PAMr_nr_mas_04_4 G2
PA14_70580	Two-component system	PAMr_nr_mas_01_2 A11
PA14_70750 (*phoB*) (1)	Two-component system	ExMr_nr_mas_01_2 C7
PA14_70750 (*phoB*) (2)	Two-component system	PAMr_nr_mas_12_3 A6
PA14_70760 (*phoR*)	Two-component system	PAMr_nr_mas_09_2 C1
PA14_70790	Two-component system	PAMr_nr_mas_08_4 D1
PA14_70800 (*phoU*)	Transcriptional regulator	PAMr_nr_mas_01_4 C5
PA14_70860	Two-component system	PAMr_nr_mas_05_1 D10
PA14_70970 (*betI*)	Transcriptional regulator	PAMr_nr_mas_10_3 G8
PA14_71070	Transcriptional regulator	PAMr_nr_mas_01_3 G2
PA14_71090	Transcriptional regulator	PAMr_nr_mas_08_3 H3
PA14_71200	Translation initiation inhibitor	PAMr_nr_mas_05_2 H3
PA14_71640 (*hypT*)	Transcriptional regulator	PAMr_nr_mas_04_2 D2
PA14_71680 (1)	Transcriptional regulator	ExMr_nr_mas_01_2 C8
PA14_71680 (2)	Transcriptional regulator	PAMr_nr_mas_04_3 E5
PA14_71750	Transcriptional regulator	PAMr_nr_mas_12_1 B1
PA14_71780	Transcriptional regulator	PAMr_nr_mas_02_4 G8
PA14_72380 (*algB*)	Two-component system	PAMr_nr_mas_01_3 B9
PA14_72390	Two-component system	PAMr_nr_mas_07_1 D4
PA14_72420 (1)	Two-component system	PAMr_nr_mas_05_4 H11
PA14_72420 (2)	Two-component system	PAMr_nr_mas_15_1 E11
PA14_72510 (*thrB*)	Serine/Threonine kinase	PAMr_nr_mas_03_3 C11
PA14_72560 (*np20*)	Transcriptional regulator	PAMr_nr_mas_10_3 C3
PA14_72650	Transcriptional regulator	PAMr_nr_mas_04_1 F2
PA14_72690 (1)	Two-component system	PAMr_nr_mas_02_1 D9
PA14_72690 (2)	Two-component system	PAMr_nr_mas_10_1 H10
PA14_72720	Transcriptional regulator	PAMr_nr_mas_10_4 B6
PA14_72740	Two-component system	PAMr_nr_mas_10_3 H7
PA14_72760	Two-component system	PAMr_nr_mas_03_2 F12
PA14_72850	Two-component system	PAMr_nr_mas_11_3 B4

PA14_72890	Transcriptional regulator	PAMr_nr_mas_02_4 D11
PA14_73020	Two-component system	PAMr_nr_mas_05_3 F3
PA14_73190 (*glmR*) (1)	Transcriptional regulator	PAMr_nr_mas_03_3 H4
PA14_73190 (*glmR*) (2)	Transcriptional regulator	PAMr_nr_mas_07_1 D6
PA14 transposon insertion mutant library ***Other mutants***	**PAMr: PA14 wild-type background, MAR2xT7 transposon, Gm^r**	**M. Ausubel, Harvard (4)**
PA14_32400 (*mexE*)	RND multidrug efflux membrane protein	PAMr_nr_mas_13_3 E11
PA14_32390 (*mexF*)	RND multidrug efflux transporter	PAMr_nr_mas_07_1 B11
PA14_32380 (*oprN*)	RND multidrug efflux outer membrane protein	PAMr_nr_mas_13_4 B8
PA14_60850 (*mexC*) (1)	RND multidrug efflux membrane protein	PAMr_nr_mas_01_4 H2
PA14_60850 (*mexC*) (2)	RND multidrug efflux membrane protein	PAMr_nr_mas_15_1 H5
PA14_60830 (*mexD*) (1)	RND multidrug efflux transporter	PAMr_nr_mas_03_1 A9
PA14_60830 (*mexD*) (2)	RND multidrug efflux transporter	PAMr_nr_mas_12_3 A1
PA14_60820 (*oprJ*)	RND multidrug efflux outer membrane protein	PAMr_nr_mas_12_1 D6
PA14_05310 (*gshB*)	Glutathione synthetase	PAMr_nr_mas_10_3 E9
PA14_05300 (*tonB3*) (1)	TonB-domain containing protein	PAMr_nr_mas_04_4 B5
PA14_05300 (*tonB3*) (2)	TonB-domain containing protein	PAMr_nr_mas_15_2 G8
PA14_07355 (*rclX*)	Predicted peroxiredoxin	PAMr_nr_mas_02_4 H11
PA14_70390 (*crc*)	Catabolite repression control protein	PAMr_nr_mas_11_1 E7
Pseudomonas aeruginosa		
Δ*rclR*	Δ*rclR* in-frame deletion in the PA14 background	This study
Δ*rclX*	Δ*rclX* in-frame deletion in the PA14 background	This study
Δ*pyeRM-xenB*	Δ*pyeRM-xenB* in-frame deletion in the PA14 background	This study
Δ*oxyR*	Δ*oxyR* in-frame deletion in the PA14 background	This study
Clinical 1	*P. aeruginosa* clinical CF isolate	Royal Brompton Hospital
Clinical 2	*P. aeruginosa* clinical CF isolate	Royal Brompton Hospital
Clinical 3	*P. aeruginosa* clinical CF isolate	Royal Brompton Hospital
E. coli		
DH5α	Commercial chemically competent cloning strain	Invitrogen
CC118λpir	Host strain for pKNG101 replication Δ(*ara-leu*) *araD* Δ*lacX74 galE galK phoA20 thi-1 rpsE rpoB argE*(Am) *recA1* Rf^r (λpir)	K. N. Timmis, Technical University of Braunschweig, Germany (5)
**Plasmid**	**Description**	**Source**
pCR™-Blunt	Cloning vector for blunt-end DNA fragments, *lac* promoter, *lacZ*α- *ccdB* fusion gene, T7 promoter, pUC origin, Zeocin^r, Km^r	Invitrogen
pRK2013	Helper plasmid for triparental mating, with *tra* functions, ColE1 replicon, Km^r	(6)

pKNG101	Suicide vector in *P. aeruginosa*, *sacB*, *oriR6K*, *mobRK2*, Sm^r	(7)
rclR-pKNG101	PA14 *rclR* mutator fragment cloned into the BamHI site in pKNG101 suicide vector, Sm^r	This study
rclX-pKNG101	PA14 *rclX* mutator fragment cloned into the BamHI site in pKNG101 suicide vector, Sm^r	This study
pyeRM-xenB-pKNG101	PA14 *pyeRM-xenB* mutator fragment cloned into the BamHI site in pKNG101 suicide vector, Sm^r	This study
oxyR-pKNG101	PA14 *oxyR* mutator fragment cloned into the XbaI site in pKNG101 suicide vector, Sm^r	This study
pUCP18	Broad-host-range, *E. coli* – *Pseudomonas* shuttle vector, Ap^r/ Cb^r	(8)
rclR-pUCP18	PA14 promoter region and ORF of *rclR* cloned into the HindIII/XbaI sites in pUCP18, Ap^r/ Cb^r	This study
rclX-pUCP18	PA14 promoter region and ORF of *rclX* cloned into the HindIII/XbaI sites in pUCP18, Ap^r/ Cb^r	This study
pMP220	Broad-host-range, promoter-probe vector; IncP replicon*,* *lacZ*, Tc^r	(9)
rclR-lacZ	PA14 promoter region (117 bp) of *rclR* cloned into the EcoRI/PstI sites upstream of the promoterless *lacZ* gene in pMP220, Tc^r	This study
rclX-lacZ	PA14 promoter region (117 bp) of *rclX* cloned into the EcoRI/PstI sites upstream of the promoterless *lacZ* gene in pMP220, Tc^r	This study
**Primer**	**Sequence 5' to 3'**	**Target and direction**
Primers for constructing deletion mutants		
rclR-1F	GCGGGATCCGAACTGGTCGTAGG CTTCC	5' region upstream of *rclR*, forward
rclR-2R	TCAGCGAGCTGGGTCCATGGGAT GAGC	3' region upstream of *rclR*, reverse
rclR-3F	ATGGACCCAGCTCGCTGAAAGCG CTAC	5' region downstream of *rclR*, forward
rclR-4R	GCGGGATCCTTCGGGTTCTGGCCG TAC	3' region downstream of *rclR*, reverse
rclR-5F	GAGTTTCGCCCAGTACACC	5' region outside of *rclR*, forward
rclR-6R	GGAAATCAGTGCGTCGATGG	3' region outside of *rclR*, reverse
rclX-1F	GCGGGATCCGTAGCTGCCGCAAA GCATG	5' region upstream of *rclX*, forward
rclX-2R	TCACTTTTTGTTCAGCATGGTGAT ATTCTCC	3' region upstream of *rclX*, reverse
rclX-3F	ATGCTGAACAAAAAGTGAGGCCG TACAGG	5' region downstream of *rclX*, forward
rclX-4R	GCGGGATCCAACATCCATGTCCG	3' region downstream of

	ACCTG	*rclX*, reverse
rclX-5F	AAGCTGTTCCAGGGTCCAG	5' region outside of *rclX*, forward
rclX-6R	GATGCGGATCAGGTCCATG	3' region outside of *rclX*, reverse
pyeRM-xenB-1F	GCGGGATCCTTCACGCAAGACCG CGAAC	5' region upstream of *pyeRM-xenB*, forward
pyeRM-xenB-2R	TCAGGCCAGGGTCGGATACAGTG GCATGGCGGTGGT	3' region upstream of *pyeRM-xenB*, reverse
pyeRM-xenB-3F	ATGCCACTGTATCCGACCCTGGCC TGATCG	5' region downstream of *pyeRM-xenB*, forward
pyeRM-xenB-4R	GCGGGATCCCCGTGGTGCTGCTCT TCAACC	3' region downstream of *pyeRM-xenB*, reverse
pyeRM-xenB-5F	TGGCAGAGCGTTGAACGTC	5' region outside of *pyeRM-xenB*, forward
pyeRM-xenB-6R	ACCATGGCGAGCAAGTTCG	3' region outside of *pyeRM-xenB*, reverse
oxyR-1F	GCTCTAGAATGATCCGTCCGGAAT AGG	5' region upstream of *oxyR*, forward
oxyR-2R	CATGTGGCGGATGGTCTCGAGGG TCATGGCTGCTCA	3' region upstream of *oxyR*, reverse
oxyR-3F	ATGACCCTCGAGACCATCCGCCAC ATG	5' region downstream of *oxyR*, forward
oxyR-4R	GCTCTAGAGGTATTCCGGATGGTA GATC	3' region downstream of *oxyR*, reverse
oxyR-5F	TCGTCGACACCGATGTAGC	5' region outside of *oxyR*, forward
oxyR-6R	GTAATCGCGGGCCAGTTC	3' region outside of *oxyR*, reverse
***Primers for cloning rclR and rclX into* pUCP18**		
rclR-CF	CCCAAGCTTGGTGATATTCTCCGT TGCTTTC	5' promoter region and *rclR* ORF, forward
rclR-CR	GCTCTAGACTTTCAGCGAGCGGA GTC	3' promoter region and *rclR* ORF, reverse
rclX-CF	CCCAAGCTTGGGATGAGCCGATC AGGT	5' promoter region and *rclX* ORF, forward
rclX-CR	GCTCTAGAGCCTGTACGGCCTCAC TT	3' promoter region and *rclX* ORF, reverse
Primers for constructing lacZ fusion plasmids		
PrclR-F	CCGGAATTCGGTGATATTCTCCGT TGCTTTC	5' *rclR* promoter region, forward
PrclR-R	GGCTGCAGGGGATGAGCCGATCA GGTC	3' *rclR* promoter region, reverse
PrclX-F	CCGGAATTCGGGATGAGCCGATC AGGTC	5' *rclX* promoter region, forward
PrclX-R	GGCTGCAGGGTGATATTCTCCGTT GCTTTC	3' *rclX* promoter region, reverse
Primers for plasmid sequencing		
pCR™-Blunt and pUCP18 sequencing		
M13 Forward -20	Refer to pCR™-Blunt sequence	5' region within pCR™-

	(Invitrogen) and pUCP18 sequence, (8)	Blunt and pUCP18 vectors, forward
M13 Reverse	Refer to pCR™-Blunt sequence (Invitrogen) and pUCP18 sequence, (8)	3' region within pCR™-Blunt and pUCP18 vectors, reverse
lacZ plasmid sequencing		
Mid-PrclR	CGTCGGGTTACCAGTCTC	5' region in *rclR* promoter, forward
Mid-PrclX	ACTGGTCGAGACTGGTAAC	5' region in *rclX* promoter, forward
Mid-lacZ	GCACTCCAGCCAGCTTTC	3' region in *lacZ* gene, reverse
Primers for qRT-PCR		
rclR-F	CATCCCGCTGTACCAGTTGAAC	133 bp amplicon, forward
rclR-R	GGTCGACTCGTCCATATGCAC	133 bp amplicon, reverse
rclX-F	TGAACAACTGGACCGAGTTCGT	77 bp amplicon, forward
rclX-R	GAGCATCTTCGGGTGTTGCTT	77 bp amplicon, reverse
PA14_21570-F	TGAAAAAGATTTCCCTCGCTTCCT	152 bp amplicon, forward
PA14_21570-R	GGAACCTTCCTTGGCCTTCTC	152 bp amplicon, reverse
PA14_34750-F	CGGTGAGTTGCAGATCCATGT	142 bp amplicon, forward
PA14_34750-R	ATAGGAAAGGTCGGAGTGCCAG	142 bp amplicon, reverse
PA14_54120-F	CCACGCCGATGTACAACTTCA	102 bp amplicon, forward
PA14_54120-R	CGCCATTGTCGAGGACGAA	102 bp amplicon, reverse
PA14_26130-F	CGCAACCGCATCGTCATG	91 bp amplicon, forward
PA14_26130-R	GCGCATAGTAGAGAGCGTTCAG	91 bp amplicon, reverse
PA14_60490-F	CTCATCGAAAAAGGCCGCTACG	116 bp amplicon, forward
PA14_60490-R	ATGGTACCCATTGGCGTCTTCA	116 bp amplicon, reverse
rpoD-F	AGGCCGTGAGCAGGGATAC	113 bp amplicon, forward
rpoD-R	TCCCCATGTCGTTGATCATG	113 bp amplicon, reverse

TABLE S2 MexT positively-regulated genes tested for HOCl susceptibility

PA14 Locus Tag/ Gene name[a]	PAO1 Locus Tag	Protein description	Conserved MexT-binding site in regulatory region[b]	PA14 transposon mutant available[c]	HOCl-sensitive (lag increased >3 hours compared to WT)[d]
PA14_22420	PA3229	Hypothetical protein	Yes	Yes	No
PA14_22740	PA3205	Hypothetical protein	No	No	N/A
PA14_27755	PA2813	Probable glutathione *S*-transferase	Yes	Yes	Yes
PA14_27770	PA2812	Probable ATP-binding component of ABC transporter	No	Yes x2	Yes
PA14_27780	PA2811	Probable permease of ABC-2 transporter	No	Yes	No
PA14_28410	PA2759	Hypothetical protein	Yes	Yes	No
PA14_32420 *mexS*	PA2491	Probable oxidoreductase	No	No	N/A
PA14_32480	PA2486	Hypothetical protein	Yes	Yes	No
PA14_39060	PA1970	Hypothetical protein	Yes	Yes	Yes
PA14_41990	PA1744	Hypothetical protein	Yes	Yes	Yes
PA14_61180	PA4623	Hypothetical protein	Yes	No	N/A
PA14_64530	PA4881	Hypothetical protein	Yes	No	N/A

[a]Listed are 12 of the 16 genes positively-regulated by MexT (the other 4 are *mexEF-oprN* and *pyeR* genes, shown in the article) (10). [b]The conserved MexT-binding site is found upstream of 10 of these genes, 8 from the table, and *mexE* and *pyeR* (10). [c]PA14 transposon mutants were available in 8 of these 12 genes (4). [d]Of the 8 mutants, 4 displayed HOCl-sensitivity, albeit none were as sensitive as the *mexE* mutant.

REFERENCES

1. Koshkin A, Nunn CM, Djordjevic S, Ortiz de Montellano PR. 2003. The mechanism of *Mycobacterium tuberculosis* alkylhydroperoxidase AhpD as defined by mutagenesis, crystallography, and kinetics. J Biol Chem 278:29502-8.
2. Nunn CM, Djordjevic S, Hillas PJ, Nishida CR, Ortiz de Montellano PR. 2002. The crystal structure of *Mycobacterium tuberculosis* alkylhydroperoxidase AhpD, a potential target for antitubercular drug design. J Biol Chem 277:20033-40.
3. Rahme LG, Stevens EJ, Wolfort SF, Shao J, Tompkins RG, Ausubel FM. 1995. Common virulence factors for bacterial pathogenicity in plants and animals. Science 268:1899-902.
4. Liberati NT, Urbach JM, Miyata S, Lee DG, Drenkard E, Wu G, Villanueva J, Wei T, Ausubel FM. 2006. An ordered, nonredundant library of *Pseudomonas aeruginosa* strain PA14 transposon insertion mutants. Proc Natl Acad Sci U S A 103:2833-8.
5. Herrero M, de Lorenzo V, Timmis KN. 1990. Transposon vectors containing non-antibiotic resistance selection markers for cloning and stable chromosomal insertion of foreign genes in gram-negative bacteria. J Bacteriol 172:6557-67.
6. Figurski DH, Helinski DR. 1979. Replication of an origin-containing derivative of plasmid RK2 dependent on a plasmid function provided in trans. Proc Natl Acad Sci U S A 76:1648-52.
7. Kaniga K, Delor I, Cornelis GR. 1991. A wide-host-range suicide vector for improving reverse genetics in gram-negative bacteria: inactivation of the *blaA* gene of *Yersinia enterocolitica*. Gene 109:137-41.
8. Schweizer HP. 1991. *Escherichia-Pseudomonas* shuttle vectors derived from pUC18/19. Gene 97:109-21.
9. Spaink HP, Okker RJ, Wijffelman CA, Pees E, Lugtenberg BJ. 1987. Promoters in the nodulation region of the *Rhizobium leguminosarum* Sym plasmid pRL1JI. Plant Mol Biol 9:27-39.
10. Tian ZX, Fargier E, Mac Aogain M, Adams C, Wang YP, O'Gara F. 2009. Transcriptome profiling defines a novel regulon modulated by the LysR-type transcriptional regulator MexT in *Pseudomonas aeruginosa*. Nucleic Acids Res 37:7546-59.
